# Critically ill adult patients with acute leukemia: a systematic review and meta-analysis

**DOI:** 10.1186/s13613-024-01409-9

**Published:** 2025-01-16

**Authors:** Dara Chean, David Luque-Paz, Daniele Poole, Sofiane Fodil, Etienne Lengliné, Thibault Dupont, Achille Kouatchet, Michael Darmon, Élie Azoulay

**Affiliations:** 1 Medical Intensive Care Unit, Saint-Louis Teaching Hospital, Paris University, 1 Avenue Claude Vellefaux, Paris, 75010 France; 2 European Society of Intensive Care Medicine (ESICM) Methodology Group, Anderlecht, Belgium; 3 Infectious Diseases and Intensive Care Unit, Pontchaillou University Hospital, Rennes, France; 4 Operative Unit of Anesthesia and Intensive Care, S. Martino Hospital, Belluno, Italy; 5 Hematology Department, Saint-Louis Teaching Hospital, Paris, France; 6 Medical Intensive Care Unit, Angers Teaching Hospital, Angers, France

**Keywords:** Intensive care unit, Acute myeloid leukemia, Acute lymphoblastic leukemia, Hematological malignancies, Acute respiratory failure, Meta-analysis, Systematical review

## Abstract

**Background:**

To describe the use of life-sustaining therapies and mortality in patients with acute leukemia admitted to the intensive care unit (ICU).

**Methods:**

The PubMed database was searched from January 1st, 2000 to July 1st, 2023. All studies including adult critically ill patients with acute leukemia were included. Two reviewers independently selected the studies, assessed bias using the Newcastle-Ottawa scale for cohort studies, and performed data extraction from full-text reading. We performed a proportional meta-analysis using a random effects model. The primary outcome was all-cause ICU mortality. Secondary outcomes included reasons for ICU admission, use of organ support therapies (mechanical ventilation, vasopressors and renal replacement therapy), hospital, day-90 and one-year mortality rates.

**Results:**

Of the 1,331 studies screened, 136 (24,861 patients) met the inclusion criteria and were included in the meta-analysis. Acute myeloid leukemia affected 16,269 (66%) patients, acute lymphoblastic leukemia affected 835 (3%) patients, and the type of leukemia was not specified in 7,757 (31%) patients. Acute respiratory failure (70%) and acute circulatory failure (25%) were the main reasons for ICU admission. Invasive mechanical ventilation, vasopressors and renal replacement therapy, were needed in 65%, 53%, and 23% of the patients, respectively. ICU mortality was available in 51 studies (6,668 patients, of whom 2,956 died throughout their ICU stay), resulting in a metanalytical proportion of 52% (95% CI [47%; 57%]; *I*^2^ 93%). In a meta-regression, variables that influenced ICU mortality included year of publication, and intubation rate.

**Conclusion:**

Acute respiratory failure is the main reason for ICU admission in patients with acute leukemia. Mechanical ventilation is the first life-sustaining therapy to be used, and also a strong predictor of mortality.

**Trial registration:**

This study’s protocol was preregistered on PROSPERO (CRD42023439630).

**Supplementary Information:**

The online version contains supplementary material available at 10.1186/s13613-024-01409-9.

## Background

Acute leukemia is a life-threatening hematologic malignancy characterized by a bone marrow infiltration from a clonal proliferation of poorly differentiated hematopoietic progenitors: myeloblasts in acute myeloid leukemia (AML) or lymphoblasts in acute lymphoblastic leukemia (ALL). Every year in the USA, about 30,000 new diagnoses of acute leukemia are made [1].

Impressive advances were made over the last two decades to establish new disease classification, rely on molecular biology and cytogenetic factors to update prognostic factors, as well as to reshape molecular disease pathways for both the diagnostic and the therapeutic approach [2]. These improvements have led to an increased survival of patients with acute leukemia [3]. For instance, studies have reported a decline in death rates due to leukemia from 7.7 to 5.8 per 100,000 persons between 2000 and 2020 [4].

The aging population is also exposed to the diagnosis of acute leukemia, as clonal hematopoiesis is more prevalent in elderly patients [5]. Thus, the number of patients that are susceptible of developing life-threatening condition related to infection, to the underlying acute leukemia (lung infiltration or leukostasis) or drug-related toxicity has also increased [6].

Admission to intensive care unit (ICU) is required in up to 25% of patients diagnosed with AML throughout the disease [7, 8]. Advances in the understanding of organ dysfunction in patients with leukemia and improved standard of care for the critically ill resulted in decreased mortality over the last two decades [9, 10]. Furthermore, critically ill acute leukemia patients discharged alive from the ICU exert comparable long-term survival and complete remission rates than those never admitted to the ICU [11, 12].

The large number of observational single-center studies published over the last decades describe a skewed view of who actually are the patients with acute leukemia admitted to the ICU. However, these published data are biased by the type of published patients, the large timeframe of this literature, the predominance of high-volume centers, and by the predominance of studies describing specific complications (i.e., tumor lysis syndrome, acute respiratory failure, etc.). We sought to provide a contemporary epidemiological picture of adult patients with acute leukemia admitted to the ICU.

## Methods

### Registration

This study’s protocol was preregistered on PROSPERO (CRD42023439630). Institutional review board approval was not required, as this study did not include individual patient data. This study followed the Preferred Reporting Items for Systematic Reviews and Meta-Analyses (PRISMA) reporting guidelines (Table S1).

### Search strategy

As the last two decades are often described as a time of major changes in the management of critically ill immunocompromised patients, resulting in improved survival, the PubMed database was systematically searched from January 1st, 2000 to July 1st, 2023.

We searched for studies with a combination of the following index terms: ((“leukemia“[MeSH Terms]) OR (“hematologic neoplasms“[MeSH Terms]) OR (“hematologic diseases“[MeSH Terms])) AND ((“intensive care units“[MeSH Terms]) OR (“critical care“[MeSH Terms])). Studies including the following index terms were excluded before screening: (“child“[MeSH Terms]) OR (“blood transfusion“[MeSH Major Topic]) OR (“blood coagulation“[MeSH Major Topic]). Studies registered as reviews or meta-analyses were excluded.

### Selection criteria

Two reviewers (DC and DLP) independently screened titles and abstracts of publications that were identified through the systematical search conducted on PubMed. We excluded studies that: [1] were neither in English or French [2], enrolled neonatal and pediatric patients (< 18 years of age) [3], were conducted in a non-ICU setting [4], did not enroll patients with acute leukemia [5], enrolled patient with non-malignant hematologic disease [6], enrolled exclusively patients that underwent hematologic stem cell transplantation (HSCT). A third independent reviewer (EA) solved the disagreements during the screening phase.

### Data extraction and outcomes

Two reviewers (DC and DLP) independently assessed full texts of the selected publications for data extraction. A pre-designed data extraction form was used to collect the following variables: author information, publication year, studies’ region of origin, period of data collection, number of patients, number of patients with AML, number of patients with ALL, main demographic characteristics (mean age, sex ratio, severity scores at ICU admission) and outcomes. The primary outcome was ICU mortality. The secondary outcomes were reasons for ICU admission, intubation rate, use of vasopressors and renal replacement therapy, hospital mortality, day-90 mortality and 12-month mortality.

Outcomes for patients with AML or ALL were collected separately if possible. When the type of acute leukemia was not specified, patients were assigned to the group of undifferentiated leukemia.

### Quality assessment

The application of our search strategy and inclusion criteria resulted in including only observational studies. No randomized studies were included in this systematic review. Quality assessment of observational studies was performed by two independent reviewers (DC and DLP) using the Newcastle-Ottawa Scale for cohort studies [13]. Reviewers resolved disagreements through discussion.

### Statistical analysis

Proportion estimates were calculated by pooling the study-specific estimates using random effects meta-analysis.

Statistical heterogeneity was explored using Cochran’s Q test (with *p* < 0.05 indicating that true proportions from the included studies’ populations are significantly different), *I*^2^ value (with values > 75% indicating that a considerable amount of total variance is due to between-study heterogeneity), and τ^2^ value which is the variance of the true proportions [14].

The DerSimonian-Laird estimator was used to calculate the variance τ^2^ and Knapp-Hartung adjustments were applied to calculate the confidence interval around the pooled proportion.

Proportions were compared with the use of a Wald-type test.

A pre-planned subgroup analysis was conducted to assess ICU mortality of the different types of acute leukemia (AML, ALL or undifferentiated leukemia).

We performed a multivariable meta-regression to assess the impact on ICU mortality of patients’ mean age, publication year, intubation rate, studies’ geographic area and type of acute leukemia (undifferentiated leukemia was used as reference). We identified geographic areas based on the Standard Country or Area Codes for Statistical Use (Series M No. 49, Rev.3) published by the United Nation Statistics Division and combined them into seven homogeneous regions: [1] Northern America (used as reference) [2], South America [3], Western and Northern Europe [4], Eastern Europe [5], Australia [6], Northern Africa and Western Asia [7], Eastern Asia. Predictors were predefined on their clinical relevance and were added to the meta-regression model using a forced entry method. Within the meta-regression, the DerSimonian-Laird estimator was used to calculate the heterogeneity variance τ^2^ and Knapp-Hartung adjustments were applied to calculate the confidence interval.

Four unplanned meta-regression models were run to measure changes in mechanical ventilation rate, SOFA, SAPS2, and APACHE2 severity scores by year of publication.

All tests were two-sided, with *p* < 0.05 considered statistically significant. All analyses were performed using R statistical software (version 4.1.2) and the metafor package [15].

## Results

### Characteristics of included studies

**Fig. 1:**
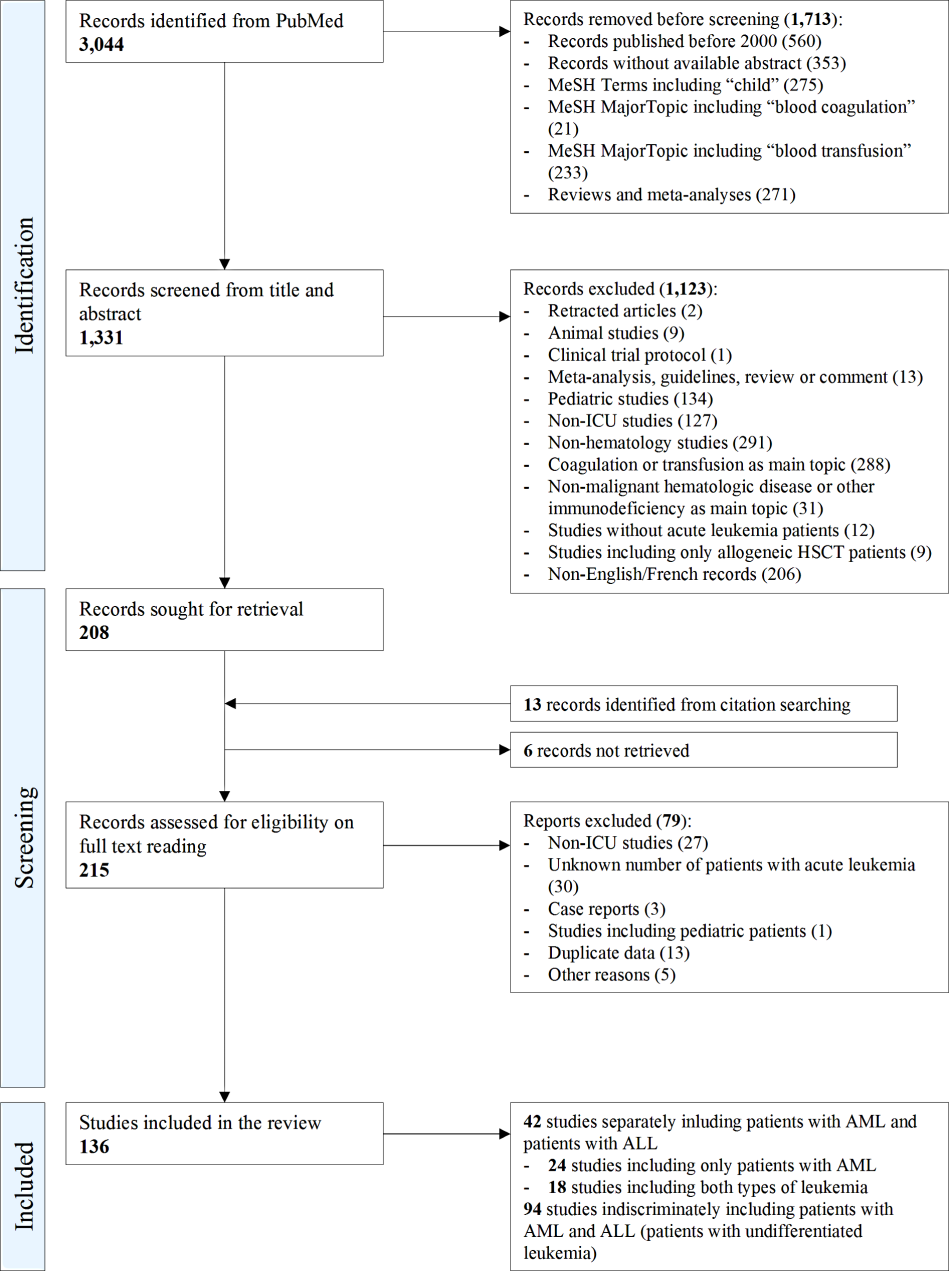
Flow diagram of study retrieval and identification ALL: acute lymphoblastic leukemia; AML: acute myeloid leukemia; HSCT: hematopoietic stem cell transplantation; ICU: intensive care unit

The search strategy identified 3,044 records, of which 1,331 were screened from title and abstract. Two hundred and eight studies were selected and were sought for retrieval. Thirteen other studies were added from citation search. In total, we reviewed the full-texts of 215 studies. As shown in Fig. 1, 136 studies including a total of 24,861 patients with acute leukemia met the inclusion criteria and were included in the meta-analysis. Twenty-four studies included only patients diagnosed with AML (*n* = 12,844). Eighteen studies included both types of leukemia (3,425 patients with AML and 835 patients with ALL). Ninety-four studies indiscriminately included patients with AML and with ALL (7,757 patients with undifferentiated leukemia).

The complete summary of study retrieval and identification is shown in Fig. 1. The main demographic variables and measured outcomes of each included study are presented in Table S2. Quality assessment using the Newcastle-Ottawa scale for cohort studies is shown in Table S3.

### Primary outcome: ICU mortality

**Fig. 2:**
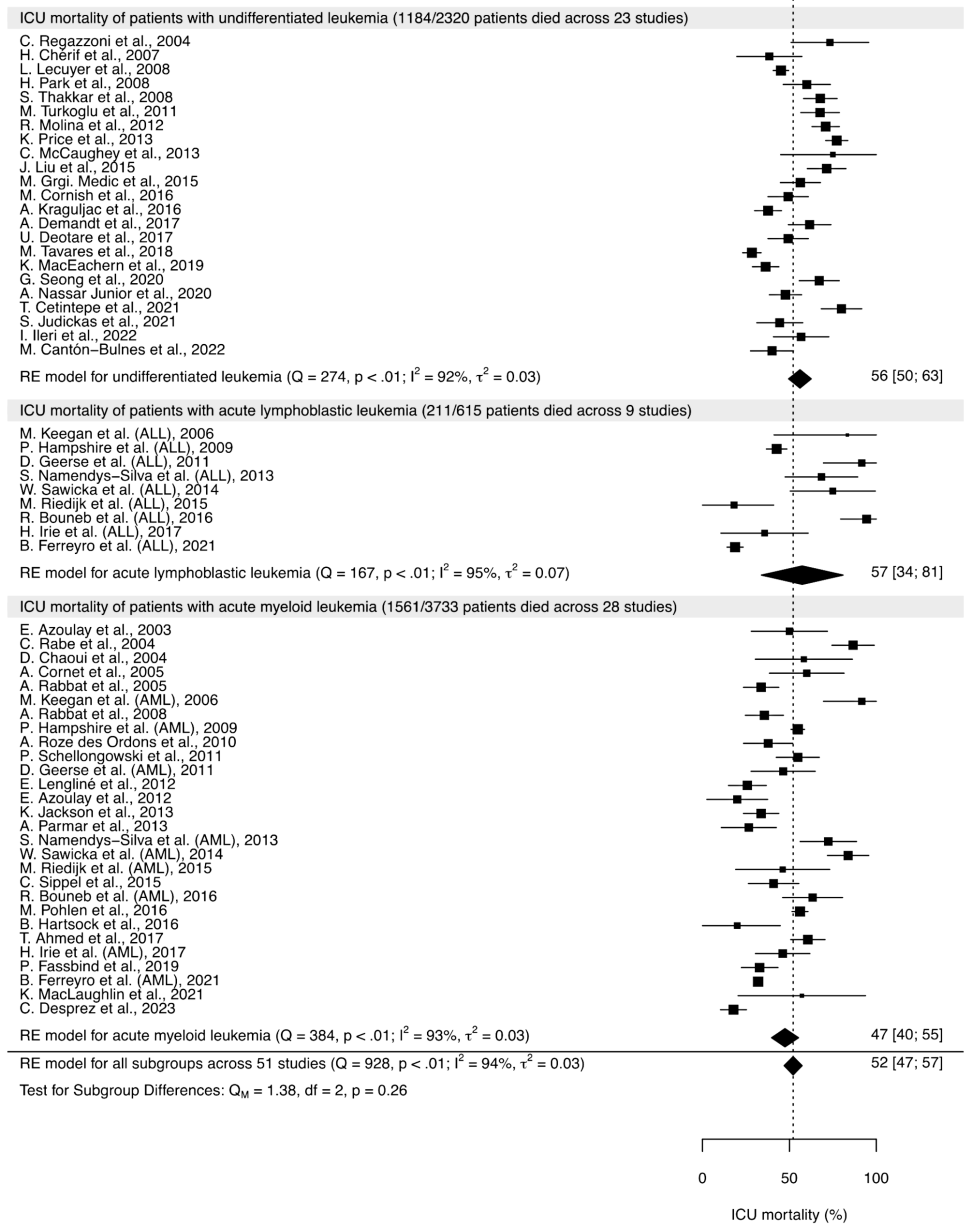
Forest plots for intensive care unit (ICU) mortality with subgroup analysis. The dotted line represents the overall ICU mortality (52%). Test for subgroup differences is a Wald-type test comparing mortality of patients with undifferentiated leukemia, acute lymphoblastic leukemia and acute myeloid leukemia ALL: acute lymphoblastic leukemia; AML: acute myeloid leukemia; ICU: intensive care unit; RE: random effects

Across 51 studies totaling 6,668 patients with acute leukemia, 2,956 died during their ICU stay. The metanalytical estimate of the overall proportion was 52% (95% CI [47%; 57%]; *I*^2^ 94%). Results of the meta-analysis and its subgroup analysis according to the type of acute leukemia are summarized in Fig. 2.

Pooled ICU mortality rates were 56% (95% CI [50%; 63%]; *I*^2^ 92%), 57% (95% CI [34%; 61%]; *I*^2^ 95%) and 47% (95% CI [40%; 55%]; *I*^2^ 93%) for patients with undifferentiated leukemia, patients with ALL and patients with AML, respectively. The *p*-value for the interaction test was 0.26 (Fig. 2).

The meta-regression included 24 studies which reported ICU mortality, patients’ mean age, publication year, intubation rate, studies’ geographic area and type of acute leukemia. Recent publication year was inversely related to ICU mortality. A direct linear relationship was found with mechanical ventilation rate (Table 1). The covariates included in this meta-regression model explained 34% of the total variance (R^2^ value). Other meta-regression-associated measurements are shown in Table S4.

**Fig. 3:**
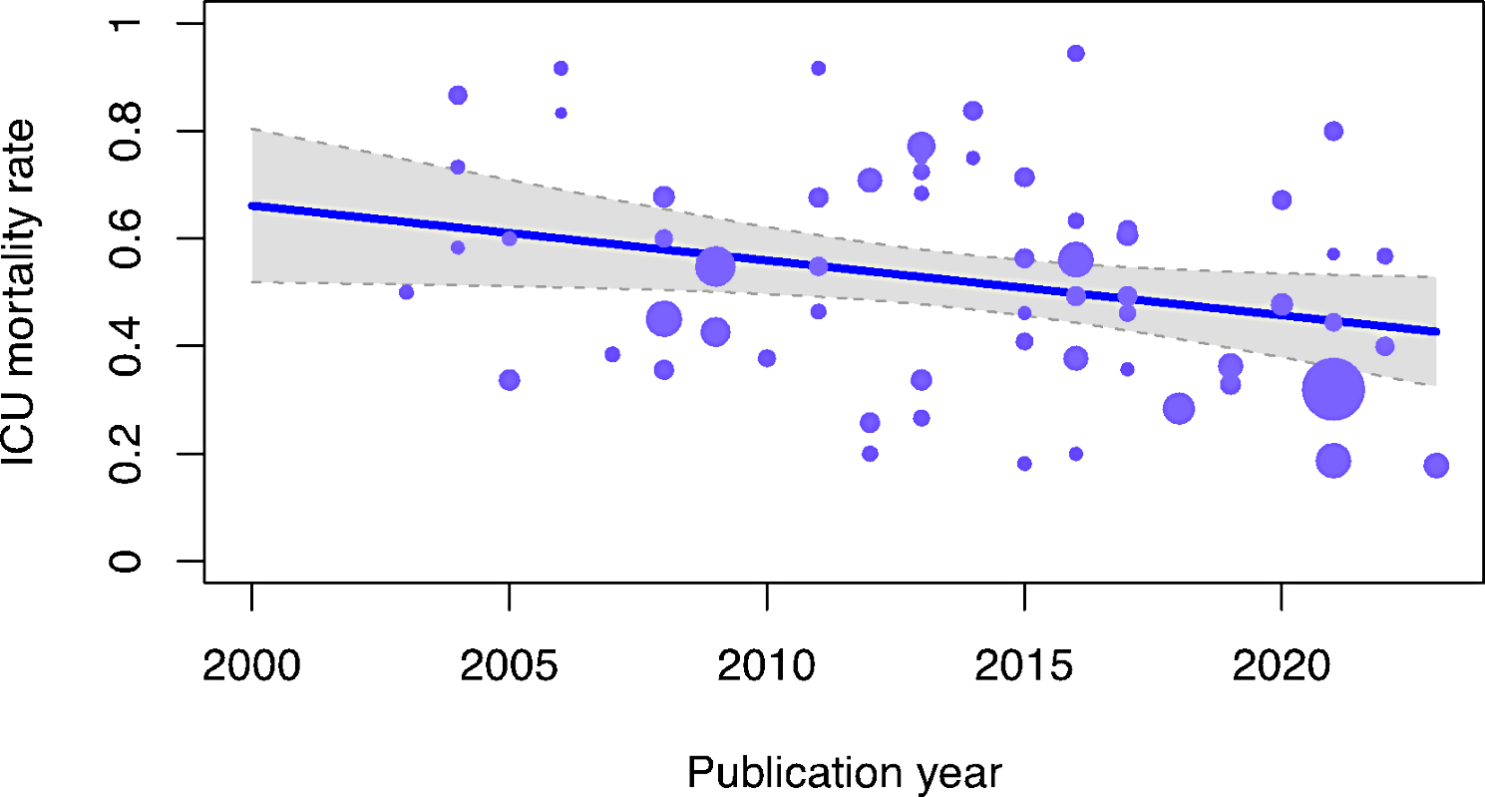
Changes of intensive care unit (ICU) mortality over time. This bubble plot shows the values of ICU mortality according to publication year. Each point represents one of the studies included in the meta-analysis. The size of each point is correlated to the sample size of each study. The blue line represents the regression line with its 95% confidence interval in grey

Figure 3 shows the changes in ICU mortality over time.

Fig. S1 shows the changes in mechanical ventilation rate, SOFA, SAPS2, and APACHE2 scores over time. The *p*-values for tests of moderators were > 0.05.

**Table 1:** Meta-regression results. A positive estimate indicates that the variable is associated with higher intensive care unit (ICU) mortality. Asterisks highlight variables that significantly contribute to ICU mortality

	Odds ratio	95% CI, lower bound	95% CI, upper bound	*p*-value
Mean patients’ age	1.05	0.99	1.12	0.08
Publication year	0.93	0.88	0.99	0.03*
Mechanical ventilation rate	1.02	1.01	1.04	< 0.01*
**Studies’ region of origin (reference: Northern America**,** k = 10)**				
Eastern Asia, k = 1	0.96	0.34	2.73	0.94
Eastern Europe, k = 1	0.83	0.27	2.56	0.72
Western and Northern Europe, k = 12	1.08	0.60	1.92	0.79
Australia, k = 1	0.82	0.29	2.30	0.68
**Type of acute leukemia (reference: undifferentiated leukemia)**				
ALL	2.45	0.66	9.04	0.16
AML	0.74	0.41	1.32	0.29

ALL: acute lymphoblastic leukemia; AML: acute myeloid leukemia; CI: confidence interval; k: number of studies

### Reason for ICU admission

Across 30 studies totaling 2,498 patients with acute leukemia, 1,652 were admitted to the ICU for acute respiratory failure (70%, 95% CI [58%; 81%]; *I*^2^ 100%) (Figure S2). Admission to the ICU for acute circulatory failure (including septic shock and cardiac arrest) concerned 491/1,561 patients across 23 studies (25%, 95% CI [13%; 37%]; *I*^2^ 100%) (Figure S2). Pooled proportions calculated from the random effects model ranged between 2 and 11% for other reasons for ICU admission (renal failure, neurologic failure, bleeding, monitoring and other reasons) (Figure S3).

### Use of life sustaining therapies

**Fig. 4:**
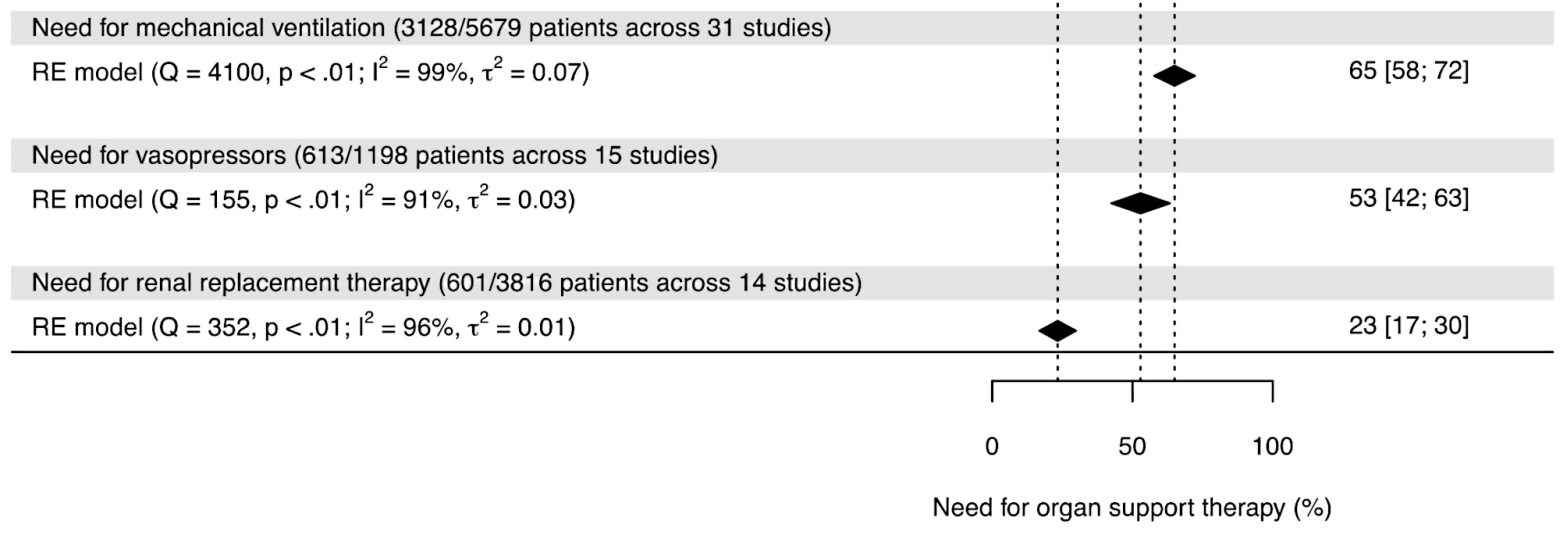
Forest plots representing the use of organ support therapies (mechanical ventilation, vasopressors and renal replacement therapy). The included studies are not displayed for visibility reasons. RE denotes for random effects

Throughout their ICU stay, 3,128/5,679 patients across 31 studies received intubation and invasive mechanical ventilation (65%, 95% CI [58%; 72%]; *I*^2^ 99%), 613/1,198 patients across 15 studies required vasopressors (53%, 95% CI [42%; 63%]; *I*^2^ 91%), and 601/3,816 patients across 14 studies underwent renal replacement therapy (23%, 95% CI [17%; 30%]; *I*^2^ 96%) (Fig. 4).

### Hospital mortality, day-90 mortality and one-year mortality

The metanalytical estimate of hospital mortality across 26 studies was 53% (7,096/15,757 patients died) (95% CI [47%; 60%]; *I*^2^ 95%) (Figure S4).

Day-90 and one-year mortality were 59% (95% CI [48%; 69%]; *I*^2^ 75%) (Figure S5) and 68% (95% CI [63%; 73%]; *I*^2^ 78%) (Figure S6), across 7 and 18 studies (357/674 and 1,688/2,477 patients died), respectively.

## Discussion

With a steadily increase of both incidence and at-risk population, and a decline of mortality over time, patients with acute leukemia are expected to use higher ICU resources in the years to come [1]. Updating clinician’s knowledge about critically ill acute leukemia patients, and understanding targets for improving outcomes are warranted. This systematic review and meta-analysis of 136 studies totaling 24,861 patients with acute leukemia reports a pooled ICU mortality rate of 52%. Acute respiratory failure is the first reason for ICU admission and mechanical ventilation the first life-sustaining therapy used. Targets for further improving survival are to be identified, especially in patients with acute respiratory failure.

Pulmonary involvement in patients with acute leukemia has attracted a lot of attention over the last years [16, 17]. At the earliest stage, leukemic infiltration and pulmonary leukostasis are highly prevalent in patients with hyperleukocytic AML, or those with monoblastic components [18]. Pulmonary tumor lysis syndrome might also occur as the consequence of leukemic burden on the lung vasculature [19]. At a latest stage, immune defects from either the leukemia or the treatments lead to severe bacterial or opportunistic infections affecting primarily the lungs. Last, drug-related pulmonary toxicity exposes leukemic patients to additional respiratory burden. Preventing acute respiratory failure in patients with acute leukemia would require a multi-level strategy that would be difficult to personalize to every patient. However, clinician’s ability to prevent intubation in hematology patients with acute respiratory failure remains controversial. Two trials of noninvasive ventilation in immunocompromised patients have reported the lack of clinical benefit [20, 21]. Moreover, a large randomized multicenter controlled trial of high-flow nasal oxygen in immunocompromised patients did not report significant reduction in intubation or mortality rates [22]. Future research will need to finetune pulmonary protective strategies in patients with acute leukemia.

Looking back at the results of this meta-analysis in patients with acute leukemia, acute respiratory failure was the most frequent reason of admission in the ICU (70% of patients), in line with previous reports [16, 23]. Moreover, the higher was the proportion of intubated patients, the higher was ICU mortality. This is in agreement with previous findings about mechanical ventilation being one of the most important prognosis factors in critically ill immunocompromised patients [23]. Pooled intubation rate was 65% in this study. However, some studies exclusively included patients with acute respiratory failure, patients under mechanical ventilation or patients with acute respiratory distress syndrome [18, 24–37]. Other organ support therapies (vasopressors and renal replacement therapy) were also frequently used.

It is noteworthy to point out the heterogeneity across studies, which might be ascribed to changes in outcomes in leukemia patients overall, including those in the ICU. This is consistent with other studies reporting a decreased mortality over time in less selected populations of immunocompromised patients [9, 38, 39]. Geographical variations did not impact ICU mortality but inferences are difficult to make as there is no representative sample from each region.

Even though this study is, to the best of our knowledge, the first meta-analysis focusing on clinical outcomes of patients with acute leukemia admitted to the ICU, it has some limitations. Only the PubMed database was searched. This did not result in significant differences in the estimate of ICU mortality, according to an unplanned sensitivity analysis that we performed secondarily, including two additional articles from a systematic search of Embase (Figure S7). Many data that might be relevant were missing from the included studies such as the frailty score, the timing between acute leukemia diagnosis and ICU admission, severity scores at admission, the presence of neutropenia, the cytogenetic characteristics of the leukemia and the withdrawing of life-sustaining treatments. Although studies focusing on HSCT were excluded, it is acknowledged that some HSCT patients may be included in the remaining studies. This may limit the conclusions that can be drawn about our target population, as the outcomes of these patients, who could not be analyzed separately, may differ from those of the general acute leukemia population. The aggregated nature of our analysis makes inferences and explanations about ICU mortality rate difficult to formulate. Without an individual patient data meta-analysis, our results must only be considered as observational findings. For instance, we do not know with certainty whether the observed decrease in mortality over time is due to changes in case mix, better selection of patients admitted to the ICU, or improvements in the quality of care for critically ill patients. However, an unplanned meta-regression analysis showed no effect of time several severity scores, but this result should be treated with caution due to lack of power. In addition, the year of publication was used to account for differences in the range of inclusion periods between studies. Although this results in an overestimation of the true date of patient inclusion, we considered it a good surrogate to assess the effect of time on mortality. Statistical heterogeneity between the included studies was high to very high. Due to the observational purpose of our study and its objective to average the current literature, heterogeneity should probably be considered an opportunity to assess differences across studies rather than a methodological issue. Some outcomes may be difficult to assess because of the small number of studies reporting them. This explains why metanalytical estimates of hospital mortality and 90-day mortality are surprisingly close to estimated ICU mortality, but with significantly wider confidence intervals. Differences in post-ICU facilities and end-of-life decisions between countries may make interpretation of hospital and 90-day mortality difficult. Finally, we could not explore long-term hematologic outcomes such as progression-free survival. Further individual patient data meta-analysis and prospective studies are warranted to specify the impact of ICU hospitalization on leukemia long-term prognosis and, inversely, the impact of leukemia molecular features on ICU outcomes.

## Conclusion

To conclude, this systematic review puts forward the high mortality rate in ICU patients with acute leukemia. These findings are significant for future prospective research in this expanding population. To ensure the accuracy of sample size calculation of future randomized controlled trials in critically ill patients with acute leukemia, this contemporary estimation of clinical outcomes prevalence is essential. The forthcoming trials are justified on the grounds of improving the quality of intensive care provided to patients with acute leukemia who become critically ill.

## Electronic supplementary material

Below is the link to the electronic supplementary material.

Supplementary Material 1

## Data Availability

Not applicable.

## Abbreviations

ALL: Acute lymphoblastic leukemia
AML: Acute myeloid leukemia
CI: Confidence interval
HSCT: Hematopoietic stem cell transplantation
ICU: Intensive care unit
PRISMA: Preferred Reporting Items for Systematic Reviews and Meta-Analyses
RE: Random effects
