# Supplementary material for: Critically ill adult patients with acute leukemia: a systematic review and meta-analysis
Source: Ann Intensive Care. 2025 Jan 16;15:9. doi: 10.1186/s13613-024-01409-9 (PMC11739448; doi:10.1186/s13613-024-01409-9)
Supplement: Supplementary file 1 — Supplementary Material 1 [file 13613_2024_1409_MOESM1_ESM.doc]

Table S1 PRISMA checklist.

|  | **#** | **Checklist item** | **Reported on page #** |
| --- | --- | --- | --- |
| **TITLE** | | |  |
| Title | 1 | Identify the report as a systematic review, meta-analysis, or both. | 1 |
| **ABSTRACT** | | |  |
| Structured summary | 2 | Provide a structured summary including, as applicable: background; objectives; data sources; study eligibility criteria, participants, and interventions; study appraisal and synthesis methods; results; limitations; conclusions and implications of key findings; systematic review registration number. | 2 |
| **INTRODUCTION** | | |  |
| Rationale | 3 | Describe the rationale for the review in the context of what is already known. | 3 |
| Objectives | 4 | Provide an explicit statement of questions being addressed with reference to participants, interventions, comparisons, outcomes, and study design (PICOS). | 4 |
| **METHODS** | | |  |
| Protocol and registration | 5 | Indicate if a review protocol exists, if and where it can be accessed (e.g., Web address), and, if available, provide registration information including registration number. | 4 |
| Eligibility criteria | 6 | Specify study characteristics (e.g., PICOS, length of follow-up) and report characteristics (e.g., years considered, language, publication status) used as criteria for eligibility, giving rationale. | 4 |
| Information sources | 7 | Describe all information sources (e.g., databases with dates of coverage, contact with study authors to identify additional studies) in the search and date last searched. | 4 |
| Search | 8 | Present full electronic search strategy for at least one database, including any limits used, such that it could be repeated. | 4 |
| Study selection | 9 | State the process for selecting studies (i.e., screening, eligibility, included in systematic review, and, if applicable, included in the meta-analysis). | 5 |
| Data collection process | 10 | Describe method of data extraction from reports (e.g., piloted forms, independently, in duplicate) and any processes for obtaining and confirming data from investigators. | 5 |
| Data items | 11 | List and define all variables for which data were sought (e.g., PICOS, funding sources) and any assumptions and simplifications made. | 5 |
| Risk of bias in individual studies | 12 | Describe methods used for assessing risk of bias of individual studies (including specification of whether this was done at the study or outcome level), and how this information is to be used in any data synthesis. | 6 |
| Summary measures | 13 | State the principal summary measures (e.g., risk ratio, difference in means). | 6 |
| Synthesis of results | 14 | Describe the methods of handling data and combining results of studies, if done, including measures of consistency (e.g., I^2^) for each meta-analysis. | 6 |

**Table S2 Main demographic variables and measured outcomes in the 136 included studies.**

| First author | Publication year | Mean age | Gender | Admission time | Any severity score | Reason for admission | Mechanical ventilation | Renal replacement therapy | Vasopressors | ICU mortality | Hospital mortality | Day-30 mortality | Day-90 mortality | One-year mortality |
| --- | --- | --- | --- | --- | --- | --- | --- | --- | --- | --- | --- | --- | --- | --- |
| D. Gruson et al. | 2000 | no | no | no | no | no | no | no | no | no | no | no | no | no |
| R. Mesters et al. | 2000 | yes | no | no | no | yes | no | no | no | no | no | no | no | no |
| A. Cornet et al. | 2005 | no | no | no | no | no | no | no | no | yes | no | no | no | no |
| A. Gordon et al. | 2005 | no | no | no | no | no | no | no | no | no | no | no | no | no |
| M. Darmon et al. | 2002 | no | no | no | no | no | no | no | no | no | no | no | no | no |
| F. Kroschinski et al. | 2002 | no | no | no | no | no | no | no | no | no | no | no | no | no |
| D. Benoit et al. | 2002 | no | no | no | no | no | no | no | no | no | no | no | no | no |
| A. Halpern et al. | 2017 | yes | yes | no | no | no | no | no | no | no | no | no | no | no |
| T. Silfvast et al. | 2003 | no | no | yes | no | no | no | no | no | no | no | no | no | yes |
| A. Moreau et al. | 2014 | yes | yes | yes | yes | yes | no | no | no | no | no | yes | no | no |
| A. Parmar et al. | 2013 | no | no | no | no | yes | yes | no | yes | yes | no | no | no | no |
| C. Regazzoni et al. | 2004 | no | no | no | no | no | no | no | no | yes | no | no | no | no |
| E. Azoulay et al. | 2004 | no | no | no | no | yes | no | no | no | no | yes | no | no | no |
| D. Gruson et al. | 2004 | no | no | no | no | no | no | no | no | no | no | no | no | no |
| A. Rabbat et al. | 2005 | yes | yes | yes | yes | yes | yes | no | no | yes | no | no | no | yes |
| A. Rabbat et al. | 2008 | yes | yes | yes | yes | yes | yes | no | yes | yes | no | no | yes | no |
| A. Roze des Ordons et al. | 2010 | yes | yes | yes | yes | yes | yes | yes | yes | yes | no | yes | yes | yes |
| A. Van de Louw et al. | 2018 | yes | yes | yes | no | yes | yes | yes | yes | no | no | yes | no | no |
| J. Moran et al. | 2005 | no | no | no | no | no | no | no | no | no | no | no | no | no |
| R. Owczuk et al. | 2005 | no | no | no | no | no | no | no | no | no | no | no | no | no |
| M. Darmon et al. | 2005 | no | no | no | no | no | no | no | no | no | no | yes | no | no |
| D. Benoit et al. | 2005 | no | no | no | no | no | no | no | no | no | no | no | no | no |
| B. Ferreyro et al. | 2021 | no | no | no | no | no | yes | yes | no | yes | yes | no | no | no |
| D. Benoit et al. | 2006 | no | no | yes | no | yes | no | no | no | no | yes | no | no | no |
| B. Lamia et al. | 2006 | no | no | no | no | no | no | no | no | no | yes | no | no | no |
| H. Chérif et al. | 2007 | no | no | no | no | no | no | no | no | yes | no | yes | no | no |
| C. Ferrà et al. | 2007 | no | no | no | no | no | no | no | no | no | no | no | no | no |
| T. Merz et al. | 2007 | no | no | no | no | no | no | no | no | no | no | no | no | no |
| B. Hartsock et al. | 2016 | yes | yes | yes | no | no | yes | no | yes | yes | no | no | no | no |
| M. Adda et al. | 2008 | no | no | no | no | yes | yes | no | no | no | no | no | no | no |
| L. Lecuyer et al. | 2008 | no | no | no | no | yes | no | no | no | yes | no | no | no | no |
| D. Vandijck et al. | 2008 | no | no | no | no | no | no | no | no | no | no | no | no | no |
| H. Park et al. | 2008 | yes | yes | no | yes | yes | yes | yes | yes | yes | yes | no | no | yes |
| S. Thakkar et al. | 2008 | yes | yes | yes | yes | no | yes | no | yes | yes | yes | no | no | yes |
| C. Desprez et al. | 2023 | yes | yes | yes | yes | yes | yes | yes | yes | yes | yes | no | no | yes |
| C. Maeng et al. | 2022 | yes | yes | no | no | no | yes | yes | no | no | yes | no | no | yes |
| C. Rhee et al. | 2009 | no | no | no | no | no | no | no | no | no | no | no | no | no |
| M. Darmon et al. | 2010 | no | no | no | no | no | no | no | no | no | no | no | no | no |
| S. McGrath et al. | 2010 | no | no | no | no | no | no | no | no | no | no | no | no | no |
| S. Bokhari et al. | 2010 | no | no | no | no | no | no | no | no | no | no | no | no | no |
| M. Soares et al. | 2010 | no | no | no | no | no | no | no | no | no | no | no | no | no |
| C. Rabe et al. | 2004 | yes | yes | no | yes | yes | yes | no | yes | yes | no | no | no | no |
| C. Sippel et al. | 2015 | yes | yes | no | yes | no | yes | yes | no | yes | no | no | no | yes |
| D. Chaoui et al. | 2004 | no | no | no | no | yes | no | no | no | yes | no | no | no | no |
| E. Mariotte et al. | 2011 | no | no | no | no | no | no | no | no | no | no | no | no | no |
| P. Povoa et al. | 2011 | no | no | no | no | no | no | no | no | no | no | no | no | no |
| M. Park et al. | 2011 | no | no | no | no | no | no | no | no | no | no | no | no | no |
| M. Turkoglu et al. | 2011 | no | no | no | yes | no | no | no | no | yes | yes | no | no | no |
| D. Geerse et al. | 2011 | no | no | no | no | no | no | no | no | yes | yes | no | no | no |
| M. Legrand et al. | 2012 | no | no | no | no | no | no | no | no | no | no | no | no | no |
| Q. Hill et al. | 2012 | no | no | no | no | no | no | no | no | no | yes | no | no | no |
| D. Mokart et al. | 2012 | no | no | no | no | no | no | no | no | no | no | no | no | no |
| G. Bird et al. | 2012 | no | no | no | no | no | no | no | no | no | yes | no | no | no |
| S. Horster al al. | 2012 | no | no | no | no | no | no | no | no | no | no | no | no | no |
| D. Schnell et al. | 2012 | no | no | no | no | yes | no | no | no | no | no | no | no | no |
| R. Molina et al. | 2012 | no | no | no | no | no | no | no | no | yes | no | no | no | no |
| D. Mokart et al. | 2012 | no | no | no | no | no | no | no | no | no | yes | yes | no | no |
| C. Yeo et al. | 2012 | no | no | no | no | no | no | no | no | no | no | no | no | no |
| E. Azoulay et al. | 2002 | yes | no | yes | no | yes | no | no | no | no | yes | no | no | no |
| E. Azoulay et al. | 2003 | yes | yes | yes | yes | yes | yes | no | no | yes | no | no | no | no |
| E. Azoulay et al. | 2012 | yes | yes | yes | yes | yes | yes | no | no | yes | no | no | no | yes |
| E. Lengliné et al. | 2012 | yes | yes | yes | no | no | yes | yes | yes | yes | no |  | no | no |
| K. Price et al. | 2013 | yes | yes | no | yes | yes | yes | no | no | yes | yes | yes | no | no |
| S. Unseld et al. | 2013 | no | no | no | no | no | no | no | no | no | no | no | no | no |
| M. Turkoglu et al. | 2013 | no | no | no | no | no | no | no | no | no | no | no | no | no |
| T. Bernal et al. | 2013 | no | no | no | no | no | no | no | no | no | no | no | no | no |
| S. Oeyen et al. | 2013 | no | no | no | no | no | no | no | no | no | no | no | no | no |
| C. McCaughey et al. | 2013 | no | no | no | no | no | no | no | no | yes | no | no | no | no |
| E. van Beers et al. | 2016 | no | no | no | no | no | no | no | no | no | no | yes | no | no |
| H. Al-Dorzi et al. | 2017 | no | no | no | no | no | yes | no | no | no | no | no | no | no |
| H. Irie et al. | 2017 | no | no | no | no | no | no | no | no | yes | no | no | no | no |
| E. Azoulay et al. | 2014 | no | no | no | no | no | no | no | no | no | no | no | no | no |
| J. Khwankeaw et al. | 2014 | no | no | no | no | no | no | no | no | no | no | no | no | no |
| D. Mokart et al. | 2014 | no | no | no | no | no | no | no | no | no | no | no | no | no |
| S. Parakh et al. | 2014 | no | no | no | no | no | no | no | no | no | no | no | no | no |
| G. Aygencel et al. | 2014 | no | no | no | no | no | no | no | no | no | no | no | no | no |
| K. Jackson et al. | 2013 | yes | yes | yes | yes | yes | yes | yes | yes | yes | no | no | no | yes |
| K. MacLaughlin et al. | 2021 | yes | yes | yes | no | yes | no | no | no | yes | yes | no | no | no |
| J. Liu et al. | 2015 | no | no | no | no | no | no | no | no | yes | yes | no | no | no |
| L. Barreto et al. | 2015 | no | no | no | no | no | no | no | no | no | no | no | no | no |
| M. Grgić Medic et al. | 2015 | no | no | no | no | no | no | no | no | yes | no | no | no | no |
| M. de Oliveira et al. | 2020 | no | no | no | no | no | no | no | no | no | no | no | no | no |
| M. Keegan et al. | 2006 | yes | yes | no | yes | yes | yes | no | no | yes | no | yes | yes | yes |
| M. Pohlen et al. | 2016 | yes | yes | yes | no | no | yes | yes | no | yes | no | no | no | no |
| M. Riedijk et al. | 2015 | no | no | no | no | no | no | no | no | yes | no | no | yes | no |
| P. Fassbind et al. | 2019 | yes | yes | yes | yes | no | yes | yes | yes | yes | yes | no | no | no |
| E. Faucher et al. | 2016 | no | no | no | no | no | no | no | no | no | no | no | no | no |
| Y. Fujiwara et al. | 2016 | no | no | no | no | no | no | no | no | no | no | no | no | no |
| M. Cornish et al. | 2016 | no | no | no | no | no | no | no | no | yes | no | yes | no | yes |
| J. Reilly et al. | 2016 | no | no | no | no | no | no | no | no | no | no | no | no | no |
| A. Kraguljac et al. | 2016 | yes | yes | yes | yes | yes | yes | yes | yes | yes | no | yes | yes | yes |
| J. Greenberg et al. | 2016 | no | no | no | no | no | no | no | no | no | no | no | no | no |
| M. Freire et al. | 2016 | no | no | no | no | no | no | no | no | no | no | yes | no | no |
| P. Cornejo-Juárez et al. | 2016 | no | no | no | no | no | no | no | no | no | no | no | no | no |
| P. Hampshire et al. | 2009 | yes | yes | no | yes | no | yes | no | no | yes | yes | no | no | no |
| P. Massion et al. | 2002 | no | no | no | no | no | no | no | no | no | yes | no | no | no |
| P. Schellongowski et al. | 2011 | yes | yes | yes | yes | yes | yes | no | no | yes | yes | yes | no | no |
| P. Wohlfarth et al. | 2014 | no | no | no | no | no | no | no | no | no | no | no | no | no |
| A. Demandt et al. | 2017 | no | no | no | no | no | no | no | no | yes | no | no | no | no |
| U. Deotare et al. | 2017 | yes | yes | no | yes | yes | yes | no | no | yes | no | no | no | yes |
| L. Russell et al. | 2017 | no | no | no | no | no | no | no | no | no | no | no | no | no |
| S. Maqsood et al. | 2017 | no | no | no | no | no | no | no | no | no | no | no | no | no |
| R. Bouneb et al. | 2016 | no | no | no | no | no | no | no | no | yes | no | no | no | no |
| L. Russell et al. | 2018 | no | no | no | no | no | no | no | no | no | no | no | no | no |
| M. Tavares et al. | 2018 | yes | yes | yes | yes | yes | yes | yes | yes | yes | yes | no | yes | yes |
| S. Pastores et al. | 2018 | no | no | no | no | no | no | no | no | no | no | no | no | no |
| N. Al-Zubaidi et al. | 2018 | no | no | no | no | no | no | no | no | no | no | no | no | no |
| E. Alp et al. | 2018 | no | no | no | no | no | no | no | no | no | no | no | no | no |
| S. Namendys-Silva et al. | 2013 | no | no | no | no | no | no | no | no | yes | yes | no | no | no |
| K. MacEachern et al. | 2019 | yes | yes | no | yes | no | yes | no | no | yes | yes | no | no | no |
| M. Kondakci et al. | 2019 | no | no | no | no | no | no | no | no | no | no | no | no | no |
| P. Bauer et al. | 2019 | no | no | no | no | yes | no | no | no | no | no | no | no | no |
| S. Richards et al. | 2016 | no | no | no | no | no | no | no | no | no | no | no | no | no |
| V. Lemiale et al. | 2020 | no | no | no | no | no | no | no | no | no | no | no | no | no |
| G. Seong et al. | 2020 | no | no | no | no | yes | yes | no | no | yes | no | no | no | no |
| A. Vijenthira et al. | 2020 | no | no | no | no | no | no | no | no | no | no | no | no | no |
| E. Kalicińska et al. | 2020 | no | no | no | no | no | no | no | no | no | no | no | no | no |
| P. Asdahl et al. | 2020 | no | yes | no | no | no | no | no | no | no | no | no | no | yes |
| A. Nassar Junior et al. | 2020 | no | no | no | no | no | no | no | no | yes | no | no | no | no |
| F. Camou et al. | 2020 | no | no | no | no | no | no | no | no | no | no | no | no | no |
| C. Ravetti et al. | 2020 | no | no | no | no | no | no | no | no | no | no | no | no | no |
| S. Slavin et al. | 2019 | yes | yes | yes | no | yes | no | no | no | no | yes | no | yes | yes |
| T. Ahmed et al. | 2017 | yes | yes | yes | no | no | yes | yes | yes | yes | no | no | no | no |
| V. de Vries et al. | 2018 | no | no | no | no | no | no | no | no | no | yes | no | no | yes |
| C. Park et al. | 2021 | no | no | no | no | no | no | no | no | no | no | no | no | no |
| G. Aygencel et al. | 2021 | no | no | no | no | no | no | no | no | no | no | no | no | no |
| M. Elfassy et al. | 2021 | no | no | no | no | no | no | no | no | no | no | no | no | no |
| T. Cetintepe et al. | 2021 | no | no | no | no | no | no | no | no | yes | no | no | no | no |
| J. Lee et al. | 2021 | no | no | no | no | no | no | no | no | no | no | no | no | no |
| S. Judickas et al. | 2021 | no | no | no | no | no | no | no | no | yes | no | no | no | no |
| W. Sawicka et al. | 2014 | no | no | no | no | no | no | no | no | yes | no | no | no | no |
| I. Ileri et al. | 2022 | no | no | no | no | no | no | no | no | yes | no | no | no | no |
| A. Nassar Junior et al. | 2022 | no | no | no | no | no | no | no | no | no | yes | no | no | no |
| N. Manjappachar et al. | 2022 | no | no | no | no | no | no | no | no | no | no | no | no | no |
| M. Cantón-Bulnes et al. | 2022 | no | no | no | no | no | no | no | no | yes | no | no | no | no |
| R. Kundu et al. | 2023 | no | no | no | no | no | no | no | no | no | no | no | no | no |

Table S3 Quality assessment of the 136 included studies with the use of Newcastle-Ottawa scale for cohort studies. The thresholds provided by the NIH (National Institutes of Health, <https://www.ncbi.nlm.nih.gov/books/NBK115843/bin/appe-fm3.pdf>) were used to convert the Newcastle-Ottawa scale to AHRQ (Agency for Healthcare Research and Quality). Among the 136 included studies, 65 studies were graded as “good quality” as they had 3 or 4 stars in selection domain AND 1 or 2 stars in comparability domain AND 2 or 3 stars in outcome/exposure domain. Other 71 studies were graded as “poor quality” as they had 0 or 1 star in selection domain OR 0 stars in comparability domain OR 0 or 1 stars in outcome/exposure domain. The 136 included studies are sorted in chronological order.

|  | **Selection** | | | | **Comparability** | **Outcome** | | |
| --- | --- | --- | --- | --- | --- | --- | --- | --- |
| **Authors** | Representativeness | Selection of the non-exposed cohort | Ascertainment | Outcome not present at start |  | Assessment | Follow-up | Adequacy of follow-up |
| R. Mesters et al.(1) | 0 | * | * | * | * | 0 | 0 | 0 |
| D. Gruson et al.(2) | 0 | * | * | * | * | 0 | 0 | 0 |
| E. Azoulay et al.(3) | 0 | * | * | * | * | 0 | 0 | 0 |
| P. Massion et al.(4) | 0 | * | * | * | * | 0 | 0 | 0 |
| F. Kroschinski et al.(5) | 0 | * | * | * | * | 0 | 0 | 0 |
| M. Darmon et al.(6) | 0 | * | * | * | * | 0 | 0 | 0 |
| D. Benoit et al.(7) | * | * | * | * | * | 0 | 0 | 0 |
| E. Azoulay et al.(8) | 0 | * | * | * | * | * | * | * |
| T. Silfvast et al.(9) | 0 | * | * | * | * | 0 | 0 | 0 |
| C. Rabe et al.(10) | 0 | * | * | * | * | * | * | * |
| D. Chaoui et al.(11) | 0 | * | * | * | * | * | * | * |
| D. Gruson et al.(12) | 0 | * | * | * | * | * | * | * |
| E. Azoulay et al.(13) | * | * | * | * | * | 0 | 0 | 0 |
| C. Regazzoni et al.(14) | 0 | * | * | * | * | * | * | * |
| A. Gordon et al.(15) | 0 | * | * | * | * | 0 | 0 | 0 |
| A. Rabbat et al.(16) | * | * | * | * | * | * | * | * |
| A. Cornet et al.(17) | 0 | * | * | * | * | * | * | * |
| J. Moran et al.(18) | 0 | * | * | * | * | 0 | 0 | 0 |
| R. Owczuk et al.(19) | 0 | * | * | * | * | * | * | * |
| D. Benoit et al.(20) | * | * | * | * | * | 0 | 0 | 0 |
| M. Darmon et al.(21) | * | * | * | * | * | 0 | 0 | 0 |
| M. Keegan et al.(22) | 0 | * | * | * | * | * | * | * |
| D. Benoit et al.(23) | 0 | * | * | * | * | 0 | 0 | 0 |
| B. Lamia et al.(24) | 0 | * | * | * | * | 0 | 0 | 0 |
| C. Ferrà et al.(25) | 0 | * | * | * | * | 0 | 0 | 0 |
| H. Chérif et al.(26) | 0 | * | * | * | * | * | * | * |
| T. Merz et al.(27) | * | * | * | * | * | 0 | 0 | 0 |
| A. Rabbat et al.(28) | * | * | * | * | * | * | * | * |
| D. Vandijck et al.(29) | * | * | * | * | * | 0 | 0 | 0 |
| S. Thakkar et al.(30) | * | * | * | * | * | * | * | * |
| L. Lecuyer et al.(31) | * | * | * | * | * | * | * | * |
| M. Adda et al.(32) | * | * | * | * | * | 0 | 0 | 0 |
| H. Park et al.(33) | * | * | * | * | * | * | * | * |
| P. Hampshire et al.(34) | * | * | * | * | * | * | * | * |
| C. Rhee et al.(35) | * | * | * | * | * | * | * | * |
| A. Roze des Ordons et al.(36) | * | * | * | * | * | * | * | * |
| M. Soares et al.(37) | 0 | * | * | * | * | 0 | 0 | 0 |
| M. Darmon et al.(38) | 0 | * | * | * | * | 0 | 0 | 0 |
| S. Bokhari et al.(39) | 0 | * | * | * | * | 0 | 0 | 0 |
| S. McGrath et al.(40) | 0 | * | * | * | * | 0 | 0 | 0 |
| P. Schellongowski et al.(41) | * | * | * | * | * | * | * | * |
| D. Geerse et al.(42) | 0 | * | * | * | * | * | * | * |
| P. Povoa et al.(43) | 0 | * | * | * | * | 0 | 0 | 0 |
| M. Park et al.(44) | 0 | * | * | * | * | 0 | 0 | 0 |
| E. Mariotte et al.(45) | 0 | * | * | * | * | 0 | 0 | 0 |
| M. Torkoglu et al.(46) | * | * | * | * | * | * | * | * |
| E. Azoulay et al.(47) | 0 | * | * | * | * | * | * | * |
| E. Lengliné et al.(48) | * | * | * | * | * | * | * | * |
| M. Legrand et al.(49) | * | * | * | * | * | 0 | 0 | 0 |
| S. Horster al al.(50) | * | * | * | * | * | * | * | * |
| Q. Hill et al.(51) | * | * | * | * | * | 0 | 0 | 0 |
| D. Mokart et al.(52) | * | * | * | * | * | * | * | * |
| R. Molina et al.(53) | * | * | * | * | * | * | * | * |
| D. Schnell et al.(54) | 0 | * | * | * | * | 0 | 0 | 0 |
| C. Yeo et al.(55) | * | * | * | * | * | * | * | * |
| G. Bird et al.(56) | * | * | * | * | * | 0 | 0 | 0 |
| D. Mokart et al.(57) | * | * | * | * | * | 0 | 0 | 0 |
| A. Parmar et al.(58) | 0 | * | * | * | * | * | * | * |
| K. Jackson et al.(59) | * | * | * | * | * | * | * | * |
| K. Price et al.(60) | * | * | * | * | * | * | * | * |
| S. Unseld et al.(61) | 0 | * | * | * | * | 0 | 0 | 0 |
| M. Turkoglu et al.(62) | 0 | * | * | * | * | * | * | * |
| S. Namendys-Silva et al.(63) | 0 | * | * | * | * | * | * | * |
| C. McCaughey et al.(64) | 0 | * | * | * | * | * | * | * |
| T. Bernal et al.(65) | 0 | * | * | * | * | 0 | 0 | 0 |
| S. Oeyen et al.(66) | 0 | * | * | * | * | 0 | 0 | 0 |
| W. Sawicka et al.(67) | 0 | * | * | * | * | * | * | * |
| P. Wohlfarth et al.(68) | 0 | * | * | * | * | 0 | 0 | 0 |
| A. Moreau et al.(69) | * | * | * | * | * | 0 | 0 | 0 |
| D. Mokart et al.(70) | * | * | * | * | * | 0 | 0 | 0 |
| J. Khwankeaw et al.(71) | * | * | * | * | * | 0 | 0 | 0 |
| G. Aygencel et al.(72) | 0 | * | * | * | * | * | * | * |
| E. Azoulay et al.(73) | * | * | * | * | * | 0 | 0 | 0 |
| S. Parakh et al.(74) | 0 | * | * | * | * | 0 | 0 | 0 |
| C. Sippel et al.(75) | * | * | * | * | * | * | * | * |
| M. Riedijk et al.(76) | 0 | * | * | * | * | * | * | * |
| M. Grgić Medic et al.(77) | * | * | * | * | * | * | * | * |
| L. Barreto et al.(78) | * | * | * | * | * | 0 | 0 | 0 |
| J. Liu et al.(79) | * | * | * | * | * | * | * | * |
| M. Pohlen et al.(80) | * | * | * | * | * | * | * | * |
| E. van Beers et al.(81) | * | * | * | * | * | 0 | 0 | 0 |
| B. Hartsock et al.(82) | 0 | * | * | * | * | * | * | * |
| S. Richards et al.(83) | * | * | * | * | * | 0 | 0 | 0 |
| R. Bouneb et al.(84) | 0 | * | * | * | * | * | * | * |
| Y. Fujiwara et al.(85) | 0 | * | * | * | * | 0 | 0 | 0 |
| A. Kraguljac et al.(86) | * | * | * | * | * | * | * | * |
| M. Cornish et al.(87) | * | * | * | * | * | * | * | * |
| M. Freire et al.(88) | 0 | * | * | * | * | 0 | 0 | 0 |
| E. Faucher et al.(89) | * | * | * | * | * | 0 | 0 | 0 |
| P. Cornejo-Juárez et al.(90) | 0 | * | * | * | * | 0 | 0 | 0 |
| J. Reilly et al.(91) | * | * | * | * | * | 0 | 0 | 0 |
| J. Greenberg et al.(92) | * | * | * | * | * | 0 | 0 | 0 |
| A. Halpern et al.(93) | * | * | * | * | * | 0 | 0 | 0 |
| H. Irie et al.(94) | 0 | * | * | * | * | * | * | * |
| T. Ahmed et al.(95) | * | * | * | * | * | * | * | * |
| H. Al-Dorzi et al.(96) | 0 | * | * | * | * | 0 | 0 | 0 |
| A. Demandt et al.(97) | * | * | * | * | * | * | * | * |
| L. Russell et al.(98) | * | * | * | * | * | 0 | 0 | 0 |
| U. Deotare et al.(99) | * | * | * | * | * | * | * | * |
| S. Maqsood et al.(100) | 0 | * | * | * | * | 0 | 0 | 0 |
| A. Van de Louw et al.(101) | 0 | * | * | * | * | 0 | 0 | 0 |
| V. de Vries et al.(102) | * | * | * | * | * | 0 | 0 | 0 |
| M. Tavares et al.(103) | * | * | * | * | * | * | * | * |
| S. Pastores et al.(104) | * | * | * | * | * | 0 | 0 | 0 |
| E. Alp et al.(105) | * | * | * | * | * | * | * | * |
| N. Al-Zubaidi et al.(106) | 0 | * | * | * | * | 0 | 0 | 0 |
| L. Russell et al.(107) | 0 | * | * | * | * | 0 | 0 | 0 |
| P. Fassbind et al.(108) | * | * | * | * | * | * | * | * |
| S. Slavin et al.(109) | * | * | * | * | * | 0 | 0 | 0 |
| K. MacEachern et al.(110) | * | * | * | * | * | * | * | * |
| M. Kondakci et al.(111) | * | * | * | * | * | * | * | * |
| P. Bauer et al.(112) | * | * | * | * | * | 0 | 0 | 0 |
| M. de Oliveira et al.(113) | 0 | * | * | * | * | 0 | 0 | 0 |
| F. Camou et al.(114) | * | * | * | * | * | 0 | 0 | 0 |
| G. Seong et al.(115) | * | * | * | * | * | * | * | * |
| P. Asdahl et al.(116) | * | * | * | * | * | 0 | 0 | 0 |
| C. Ravetti et al.(117) | 0 | * | * | * | * | 0 | 0 | 0 |
| V. Lemiale et al.(118) | * | * | * | * | * | 0 | 0 | 0 |
| A. Nassar Junior et al.(119) | 0 | * | * | * | * | * | * | * |
| A. Vijenthira et al.(120) | * | * | * | * | * | 0 | 0 | 0 |
| E. Kalicińska et al.(121) | * | * | * | * | * | * | * | * |
| B. Ferreyro et al.(122) | * | * | * | * | * | * | * | * |
| K. MacLaughlin et al.(123) | 0 | * | * | * | * | * | * | * |
| T. Cetintepe et al.(124) | * | * | * | * | * | * | * | * |
| M. Elfassy et al.(125) | * | * | * | * | * | 0 | 0 | 0 |
| C. Park et al.(126) | 0 | * | * | * | * | 0 | 0 | 0 |
| G. Aygencel et al.(127) | * | * | * | * | * | 0 | 0 | 0 |
| Š. Judickas et al.(128) | * | * | * | * | * | * | * | * |
| J. Lee et al.(129) | * | * | * | * | * | * | * | * |
| C. Maeng et al.(130) | * | * | * | * | * | 0 | 0 | 0 |
| N. Manjappachar et al.(131) | * | * | * | * | * | * | * | * |
| I. Ileri et al.(132) | 0 | * | * | * | * | * | * | * |
| A. Nassar Junior et al.(133)n | 0 | * | * | * | * | 0 | 0 | 0 |
| M. Canton-Bulnes et al.(134) | * | * | * | * | * | * | * | * |
| C. Desprez et al.(135) | * | * | * | * | * | * | * | * |
| R. Kundu et al.(136) | * | * | * | * | * | * | * | * |

Table S4 Other measurements from the meta-regression model.

| τ^2^ | Estimated amount of residual heterogeneity | 0.23 |
| --- | --- | --- |
| I^2^ before meta-regression |  | 93.6%, 95% CI (91.7%; 96.3%) |
| I^2^ after meta-regression | Residual heterogeneity / unaccounted variability | 82.9%, 95% CI (52.75%; 88.0%) |
| H^2^ | Unaccounted variability / sampling variability | 5.84, 95% CI (2.1; 8.37) |
| R^2^ | Amount of heterogeneity accounted for | 34% |

Figure S1 Changes of mechanical ventilation rate and severity scores over time. This bubble plot shows the values of mechanical ventilation rate (panel A), SOFA score (panel B), SAPS2 score (panel C), and APACHE2 score (panel D) according to publication year. Each point represents a study. The size of each point is correlated to the sample size of each study. The blue line represents the regression line with its 95% confidence interval in grey. The displayed p-values corresponds to the result of a test of moderators within a meta-regression model evaluating the effect of time (publication year) on mechanical ventilation rate and severity scores.

Figure S2 Forest plots for respiratory and circulatory admissions in the intensive care unit (ICU). Included studies are not fully displayed for visibility reasons.

Figure S3 Forest plots for other reasons of admission in the intensive care unit (ICU) than respiratory or circulatory. Included studies are not fully displayed for visibility reasons.

**Figure S4 Forest plot for hospital mortality.** The dotted line represents the pooled hospital mortality (53%)

Figure S5 Forest plot for day-90 mortality. The dotted line represents the pooled day-90 mortality (59%).

Figure S6 Forest plot for one-year mortality. The dotted line represents the pooled one-year mortality (69%).

Figure S7 Forest plot for intensive care unit (ICU) mortality with subgroup analysis. The dotted line represents the pooled one-year mortality (52%). The dotted line represents the overall ICU mortality (52%). Test for subgroup differences is a Wald-type test comparing mortality of patients with undifferentiated leukemia, acute lymphoblastic leukemia and acute myeloid leukemia. Studies from Talan et al. and Costa-Correa et al. were added from a systematical search on Embase using the following research key: ‘acute leukemia’:ab,ti AND ‘intensive care’:ab,ti AND [embase]/lim NOT ([embase]/lim AND [medline]/lim) AND [2000-2024]/py

*ALL, acute lymphoblastic leukemia; AML, acute myeloid leukemia; ICU, intensive care unit; RE, random effects*

**References**

1. Mesters RM, Helterbrand J, Utterback BG, Yan B, Chao YB, Fernandez JA, et al. Prognostic value of protein C concentrations in neutropenic patients at high risk of severe septic complications. Crit Care Med. 2000 Jul;28(7):2209–16.

2. Gruson D, Hilbert G, Valentino R, Vargas F, Chene G, Bebear C, et al. Utility of fiberoptic bronchoscopy in neutropenic patients admitted to the intensive care unit with pulmonary infiltrates. Crit Care Med. 2000 Jul;28(7):2224–30.

3. Azoulay E, Darmon M, Delclaux C, Fieux F, Bornstain C, Moreau D, et al. Deterioration of previous acute lung injury during neutropenia recovery. Crit Care Med. 2002 Apr;30(4):781–6.

4. Massion PB, Dive AM, Doyen C, Bulpa P, Jamart J, Bosly A, et al. Prognosis of hematologic malignancies does not predict intensive care unit mortality. Crit Care Med. 2002 Oct;30(10):2260–70.

5. Kroschinsky F, Weise M, Illmer T, Haenel M, Bornhaeuser M, Hoeffken G, et al. Outcome and prognostic features of intensive care unit treatment in patients with hematological malignancies. Intensive Care Med. 2002 Sep;28(9):1294–300.

6. Darmon M, Azoulay E, Alberti C, Fieux F, Moreau D, Le Gall JR, et al. Impact of neutropenia duration on short-term mortality in neutropenic critically ill cancer patients. Intensive Care Med. 2002 Dec;28(12):1775–80.

7. Benoit DD, Vandewoude KH, Decruyenaere JM, Hoste EA, Colardyn FA. Outcome and early prognostic indicators in patients with a hematologic malignancy admitted to the intensive care unit for a life-threatening complication. Critical Care Medicine. 2003 Jan;31(1):104–12.

8. Azoulay E, Fieux F, Moreau D, Thiery G, Rousselot P, Parrot A, et al. Acute monocytic leukemia presenting as acute respiratory failure. Am J Respir Crit Care Med. 2003 May 15;167(10):1329–33.

9. Silfvast T, Pettilä V, Ihalainen A, Elonen E. Multiple organ failure and outcome of critically ill patients with haematological malignancy. Acta Anaesthesiol Scand. 2003 Mar;47(3):301–6.

10. Rabe C, Mey U, Paashaus M, Musch A, Tasci S, Glasmacher A, et al. Outcome of patients with acute myeloid leukemia and pulmonary infiltrates requiring invasive mechanical ventilation-a retrospective analysis. J Crit Care. 2004 Mar;19(1):29–35.

11. Chaoui D, Legrand O, Roche N, Cornet M, Lefebvre A, Peffault de Latour R, et al. Incidence and prognostic value of respiratory events in acute leukemia. Leukemia. 2004 Apr;18(4):670–5.

12. Gruson D, Vargas F, Hilbert G, Bui N, Maillot T, Mayet T, et al. Predictive factors of intensive care unit admission in patients with haematological malignancies and pneumonia. Intensive Care Med. 2004 May;30(5):965–71.

13. Azoulay É, Thiéry G, Chevret S, Moreau D, Darmon M, Bergeron A, et al. The prognosis of acute respiratory failure in critically ill cancer patients. Medicine (Baltimore). 2004 Nov;83(6):360–70.

14. Regazzoni CJ, Irrazabal C, Luna CM, Poderoso JJ. Cancer patients with septic shock: mortality predictors and neutropenia. Support Care Cancer. 2004 Dec;12(12):833–9.

15. Gordon AC, Oakervee HE, Kaya B, Thomas JM, Barnett MJ, Rohatiner AZS, et al. Incidence and outcome of critical illness amongst hospitalised patients with haematological malignancy: a prospective observational study of ward and intensive care unit based care. Anaesthesia. 2005 Apr;60(4):340–7.

16. Rabbat A, Chaoui D, Montani D, Legrand O, Lefebvre A, Rio B, et al. Prognosis of patients with acute myeloid leukaemia admitted to intensive care. Br J Haematol. 2005 May;129(3):350–7.

17. Cornet AD, Issa AI, van de Loosdrecht AA, Ossenkoppele GJ, Strack van Schijndel RJM, Groeneveld ABJ. Sequential organ failure predicts mortality of patients with a haematological malignancy needing intensive care. Eur J Haematol. 2005 Jun;74(6):511–6.

18. Moran JL, Solomon PJ, Williams PJ. Assessment of outcome over a 10-year period of patients admitted to a multidisciplinary adult intensive care unit with haematological and solid tumours. Anaesth Intensive Care. 2005 Feb;33(1):26–35.

19. Owczuk R, Wujtewicz MA, Sawicka W, Wadrzyk A, Wujtewicz M. Patients with haematological malignancies requiring invasive mechanical ventilation: differences between survivors and non-survivors in intensive care unit. Support Care Cancer. 2005 May;13(5):332–8.

20. Benoit DD, Depuydt PO, Peleman RA, Offner FC, Vandewoude KH, Vogelaers DP, et al. Documented and clinically suspected bacterial infection precipitating intensive care unit admission in patients with hematological malignancies: impact on outcome. Intensive Care Med. 2005 Jul;31(7):934–42.

21. Darmon M, Thiery G, Ciroldi M, de Miranda S, Galicier L, Raffoux E, et al. Intensive care in patients with newly diagnosed malignancies and a need for cancer chemotherapy. Crit Care Med. 2005 Nov;33(11):2488–93.

22. Keegan MT, Nygren E, Afessa B, Hogan WJ, Harrison BA. Is there a role for inhaled nitric oxide as a rescue therapy in respiratory failure associated with hematologic malignancies? Am J Hematol. 2006 Oct;81(10):729–34.

23. Benoit DD, Depuydt PO, Vandewoude KH, Offner FC, Boterberg T, De Cock CA, et al. Outcome in severely ill patients with hematological malignancies who received intravenous chemotherapy in the intensive care unit. Intensive Care Med. 2006 Jan;32(1):93–9.

24. Lamia B, Hellot MF, Girault C, Tamion F, Dachraoui F, Lenain P, et al. Changes in severity and organ failure scores as prognostic factors in onco-hematological malignancy patients admitted to the ICU. Intensive Care Med. 2006 Oct;32(10):1560–8.

25. Ferrà C, Marcos P, Misis M, Morgades M, Bordejé ML, Oriol A, et al. Outcome and prognostic factors in patients with hematologic malignancies admitted to the intensive care unit: a single-center experience. Int J Hematol. 2007 Apr;85(3):195–202.

26. Cherif H, Martling CR, Hansen J, Kalin M, Björkholm M. Predictors of short and long-term outcome in patients with hematological disorders admitted to the intensive care unit for a life-threatening complication. Support Care Cancer. 2007 Dec;15(12):1393–8.

27. Merz TM, Schär P, Bühlmann M, Takala J, Rothen HU. Resource use and outcome in critically ill patients with hematological malignancy: a retrospective cohort study. Crit Care. 2008;12(3):R75.

28. Rabbat A, Chaoui D, Lefebvre A, Roche N, Legrand O, Lorut C, et al. Is BAL useful in patients with acute myeloid leukemia admitted in ICU for severe respiratory complications? Leukemia. 2008 Jul;22(7):1361–7.

29. Vandijck DM, Benoit DD, Depuydt PO, Offner FC, Blot SI, Van Tilborgh AK, et al. Impact of recent intravenous chemotherapy on outcome in severe sepsis and septic shock patients with hematological malignancies. Intensive Care Med. 2008 May;34(5):847–55.

30. Thakkar SG, Fu AZ, Sweetenham JW, Mciver ZA, Mohan SR, Ramsingh G, et al. Survival and predictors of outcome in patients with acute leukemia admitted to the intensive care unit. Cancer. 2008 May 15;112(10):2233–40.

31. Lecuyer L, Chevret S, Guidet B, Aegerter P, Martel P, Schlemmer B, et al. Case volume and mortality in haematological patients with acute respiratory failure. Eur Respir J. 2008 Sep;32(3):748–54.

32. Adda M, Coquet I, Darmon M, Thiery G, Schlemmer B, Azoulay E. Predictors of noninvasive ventilation failure in patients with hematologic malignancy and acute respiratory failure. Crit Care Med. 2008 Oct;36(10):2766–72.

33. Park HY, Suh GY, Jeon K, Koh WJ, Chung MP, Kim H, et al. Outcome and prognostic factors of patients with acute leukemia admitted to the intensive care unit for septic shock. Leuk Lymphoma. 2008 Oct;49(10):1929–34.

34. Hampshire PA, Welch CA, McCrossan LA, Francis K, Harrison DA. Admission factors associated with hospital mortality in patients with haematological malignancy admitted to UK adult, general critical care units: a secondary analysis of the ICNARC Case Mix Programme Database. Crit Care. 2009;13(4):R137.

35. Rhee CK, Kang JY, Kim YH, Kim JW, Yoon HK, Kim SC, et al. Risk factors for acute respiratory distress syndrome during neutropenia recovery in patients with hematologic malignancies. Crit Care. 2009;13(6):R173.

36. Roze des Ordons AL, Chan K, Mirza I, Townsend DR, Bagshaw SM. Clinical characteristics and outcomes of patients with acute myelogenous leukemia admitted to intensive care: a case-control study. BMC Cancer. 2010 Sep 28;10:516.

37. Soares M, Caruso P, Silva E, Teles JMM, Lobo SMA, Friedman G, et al. Characteristics and outcomes of patients with cancer requiring admission to intensive care units: a prospective multicenter study. Critical Care Medicine. 2010 Jan;38(1):9–15.

38. Darmon M, Guichard I, Vincent F, Schlemmer B, Azoulay E. Prognostic significance of acute renal injury in acute tumor lysis syndrome. Leuk Lymphoma. 2010 Feb;51(2):221–7.

39. Bokhari SWI, Munir T, Memon S, Byrne JL, Russell NH, Beed M. Impact of critical care reconfiguration and track-and-trigger outreach team intervention on outcomes of haematology patients requiring intensive care admission. Ann Hematol. 2010 May;89(5):505–12.

40. McGrath S, Chatterjee F, Whiteley C, Ostermann M. ICU and 6-month outcome of oncology patients in the intensive care unit. QJM. 2010 Jun;103(6):397–403.

41. Schellongowski P, Staudinger T, Kundi M, Laczika K, Locker GJ, Bojic A, et al. Prognostic factors for intensive care unit admission, intensive care outcome, and post-intensive care survival in patients with de novo acute myeloid leukemia: a single center experience. Haematologica. 2011 Feb;96(2):231–7.

42. Geerse DA, Span LFR, Pinto-Sietsma SJ, van Mook WNKA. Prognosis of patients with haematological malignancies admitted to the intensive care unit: Sequential Organ Failure Assessment (SOFA) trend is a powerful predictor of mortality. Eur J Intern Med. 2011 Feb;22(1):57–61.

43. Póvoa P, Souza-Dantas VC, Soares M, Salluh JF. C-reactive protein in critically ill cancer patients with sepsis: influence of neutropenia. Crit Care. 2011;15(3):R129.

44. Park MR, Jeon K, Song JU, Lim SY, Park SY, Lee JE, et al. Outcomes in critically ill patients with hematologic malignancies who received renal replacement therapy for acute kidney injury in an intensive care unit. J Crit Care. 2011 Feb;26(1):107.e1-6.

45. Mariotte E, Schnell D, Scieux C, Agbalika F, Legoff J, Ribaud P, et al. Significance of herpesvirus 6 in BAL fluid of hematology patients with acute respiratory failure. Infection. 2011 Jun;39(3):225–30.

46. Turkoglu M, Mirza E, Tunçcan ÖG, Erdem GU, Dizbay M, Yağcı M, et al. Acinetobacter baumannii infection in patients with hematologic malignancies in intensive care unit: risk factors and impact on mortality. J Crit Care. 2011 Oct;26(5):460–7.

47. Azoulay É, Canet E, Raffoux E, Lengliné E, Lemiale V, Vincent F, et al. Dexamethasone in patients with acute lung injury from acute monocytic leukaemia. Eur Respir J. 2012 Mar;39(3):648–53.

48. Lengliné E, Raffoux E, Lemiale V, Darmon M, Canet E, Boissel N, et al. Intensive care unit management of patients with newly diagnosed acute myeloid leukemia with no organ failure. Leuk Lymphoma. 2012 Jul;53(7):1352–9.

49. Legrand M, Max A, Peigne V, Mariotte E, Canet E, Debrumetz A, et al. Survival in neutropenic patients with severe sepsis or septic shock. Crit Care Med. 2012 Jan;40(1):43–9.

50. Horster S, Stemmler HJ, Mandel PC, Mück A, Tischer J, Hausmann A, et al. Mortality of patients with hematological malignancy after admission to the intensive care unit. Onkologie. 2012;35(10):556–61.

51. Hill QA, Kelly RJ, Patalappa C, Whittle AM, Scally AJ, Hughes A, et al. Survival of patients with hematological malignancy admitted to the intensive care unit: prognostic factors and outcome compared to unselected medical intensive care unit admissions, a parallel group study. Leuk Lymphoma. 2012 Feb;53(2):282–8.

52. Mokart D, van Craenenbroeck T, Lambert J, Textoris J, Brun JP, Sannini A, et al. Prognosis of acute respiratory distress syndrome in neutropenic cancer patients. Eur Respir J. 2012 Jul;40(1):169–76.

53. Molina R, Bernal T, Borges M, Zaragoza R, Bonastre J, Granada RM, et al. Ventilatory support in critically ill hematology patients with respiratory failure. Crit Care. 2012 Jul 24;16(4):R133.

54. Schnell D, Legoff J, Mariotte E, Seguin A, Canet E, Lemiale V, et al. Molecular detection of respiratory viruses in immunocopromised ICU patients: Incidence and meaning. Respir Med. 2012 Aug;106(8):1184–91.

55. Yeo CD, Kim JW, Kim SC, Kim YK, Kim KH, Kim HJ, et al. Prognostic factors in critically ill patients with hematologic malignancies admitted to the intensive care unit. J Crit Care. 2012 Dec;27(6):739.e1-6.

56. Bird GT, Farquhar-Smith P, Wigmore T, Potter M, Gruber PC. Outcomes and prognostic factors in patients with haematological malignancy admitted to a specialist cancer intensive care unit: a 5 yr study. Br J Anaesth. 2012 Mar;108(3):452–9.

57. Mokart D, Etienne A, Esterni B, Brun JP, Chow-Chine L, Sannini A, et al. Critically ill cancer patients in the intensive care unit: short-term outcome and 1-year mortality. Acta Anaesthesiol Scand. 2012 Feb;56(2):178–89.

58. Parmar A, Richardson H, McKinlay D, Gibney RTN, Bagshaw SM. Medical emergency team involvement in patients hospitalized with acute myeloid leukemia. Leuk Lymphoma. 2013 Oct;54(10):2236–42.

59. Jackson K, Mollee P, Morris K, Butler J, Jackson D, Kruger P, et al. Outcomes and prognostic factors for patients with acute myeloid leukemia admitted to the intensive care unit. Leuk Lymphoma. 2014 Jan;55(1):97–104.

60. Price KJ, Cardenas-Turanzas M, Lin H, Roden L, Nigam R, Nates JL. Prognostic indicators of mortality of mechanically ventilated patients with acute leukemia in a comprehensive cancer center. Minerva Anestesiol. 2013 Feb;79(2):147–55.

61. Unseld S, Schuepbach RA, Maggiorini M. ICU, hospital and one year mortality of patients suffering from solid or haematological malignancies. Swiss Med Wkly. 2013;143:w13741.

62. Türkoğlu M, Erdem GU, Suyanı E, Sancar ME, Yalçın MM, Aygencel G, et al. Acute respiratory distress syndrome in patients with hematological malignancies. Hematology. 2013 May;18(3):123–30.

63. Namendys-Silva SA, González-Herrera MO, García-Guillén FJ, Texcocano-Becerra J, Herrera-Gómez A. Outcome of critically ill patients with hematological malignancies. Ann Hematol. 2013 May;92(5):699–705.

64. McCaughey C, Blackwood B, Glackin M, Brady M, McMullin MF. Characteristics and outcomes of haematology patients admitted to the intensive care unit. Nurs Crit Care. 2013 Jul;18(4):193–9.

65. Bernal T, Pardavila EV, Bonastre J, Jarque I, Borges M, Bargay J, et al. Survival of hematological patients after discharge from the intensive care unit: a prospective observational study. Crit Care. 2013 Dec 30;17(6):R302.

66. Oeyen SG, Benoit DD, Annemans L, Depuydt PO, Van Belle SJ, Troisi RI, et al. Long-term outcomes and quality of life in critically ill patients with hematological or solid malignancies: a single center study. Intensive Care Med. 2013 May;39(5):889–98.

67. Sawicka W, Owczuk R, Wujtewicz MA, Wujtewicz M. The effectiveness of the APACHE II, SAPS II and SOFA prognostic scoring systems in patients with haematological malignancies in the intensive care unit. Anaesthesiol Intensive Ther. 2014;46(3):166–70.

68. Wohlfarth P, Staudinger T, Sperr WR, Bojic A, Robak O, Hermann A, et al. Prognostic factors, long-term survival, and outcome of cancer patients receiving chemotherapy in the intensive care unit. Ann Hematol. 2014 Oct;93(10):1629–36.

69. Moreau AS, Lengline E, Seguin A, Lemiale V, Canet E, Raffoux E, et al. Respiratory events at the earliest phase of acute myeloid leukemia. Leuk Lymphoma. 2014 Nov;55(11):2556–63.

70. Mokart D, Slehofer G, Lambert J, Sannini A, Chow-Chine L, Brun JP, et al. De-escalation of antimicrobial treatment in neutropenic patients with severe sepsis: results from an observational study. Intensive Care Med. 2014 Jan;40(1):41–9.

71. Khwankeaw J, Bhurayanontachai R. Mortality correlation factors in patients with lymphoma and acute myeloid leukemia admitted into the intensive care unit at a referral center in the south of Thailand. J Med Assoc Thai. 2014 Jan;97 Suppl 1:S77-83.

72. Aygencel G, Turkoglu M, Turkoz Sucak G, Benekli M. Prognostic factors in critically ill cancer patients admitted to the intensive care unit. J Crit Care. 2014 Aug;29(4):618–26.

73. Azoulay E, Lemiale V, Mokart D, Pène F, Kouatchet A, Perez P, et al. Acute respiratory distress syndrome in patients with malignancies. Intensive Care Med. 2014 Aug;40(8):1106–14.

74. Parakh S, Piggin A, Neeman T, Mitchell I, Crispin P, Davis A. Outcomes of haematology/oncology patients admitted to intensive care unit at The Canberra Hospital. Intern Med J. 2014 Nov;44(11):1087–94.

75. Sippel C, Kim Y, Wallau A, Brossart P, Schmidt-Wolf I, Walger P. AML versus ICU: outcome of septic AML patients in an intensive care setting. J Cancer Res Clin Oncol. 2015 Sep;141(9):1645–51.

76. Riedijk M, van den Bergh WM, van Vliet M, Kusadasi N, Span LRF, Tuinman PR, et al. Characteristics and outcomes of patients with a haematological malignancy admitted to the intensive care unit for a neurological event. Crit Care Resusc. 2015 Dec;17(4):268–73.

77. Grgić Medić M, Gornik I, Gašparović V. Hematologic malignancies in the medical intensive care unit--Outcomes and prognostic factors. Hematology. 2015 Jun;20(5):247–53.

78. Barreto LM, Torga JP, Coelho SV, Nobre V. Main characteristics observed in patients with hematologic diseases admitted to an intensive care unit of a Brazilian university hospital. Rev Bras Ter Intensiva. 2015 Sep;27(3):212–9.

79. Liu J, Cheng Q, Yang Q, Li X, Shen X, Zhang L, et al. Prognosis-related factors in intensive care unit (ICU) patients with hematological malignancies: A retrospective cohort analysis in a Chinese population. Hematology. 2015 Oct;20(9):494–503.

80. Pohlen M, Thoennissen NH, Braess J, Thudium J, Schmid C, Kochanek M, et al. Patients with Acute Myeloid Leukemia Admitted to Intensive Care Units: Outcome Analysis and Risk Prediction. PLoS One. 2016;11(8):e0160871.

81. van Beers EJ, Müller MCA, Vlaar APJ, Spanjaard L, van den Bergh WM, HEMA-ICU Study Group. Haematological malignancy in the intensive care unit: microbiology results and mortality. Eur J Haematol. 2016 Sep;97(3):271–7.

82. Hartsock B, Lim MJ, Roth CG, Raptis N, Weber D, Sehgal A, et al. ICU intervention during induction chemotherapy for adult patients with newly diagnosed acute myeloid leukemia. Leuk Res. 2016;48:16–9.

83. Richards S, Wibrow B, Anstey M, Sidiqi H, Chee A, Ho KM. Determinants of 6-month survival of critically ill patients with an active hematologic malignancy. J Crit Care. 2016 Dec;36:252–8.

84. Hamdaoui Y, Bouneb R, Azouzi A, Ayachi J, Khedher A, Chouchene I, et al. Outcome and prognostic features in respiratory critically-ill patients with hematological malignancies. European Respiratory Journal [Internet]. 2015 Sep 1 [cited 2024 Apr 20];46(suppl 59). Available from: https://erj.ersjournals.com/content/46/suppl_59/PA2142

85. Fujiwara Y, Yamaguchi H, Kobayashi K, Marumo A, Omori I, Yamanaka S, et al. The Therapeutic Outcomes of Mechanical Ventilation in Hematological Malignancy Patients with Respiratory Failure. Intern Med. 2016;55(12):1537–45.

86. Kraguljac AP, Croucher D, Christian M, Ibrahimova N, Kumar V, Jacob G, et al. Outcomes and Predictors of Mortality for Patients with Acute Leukemia Admitted to the Intensive Care Unit. Can Respir J. 2016;2016:3027656.

87. Cornish M, Butler MB, Green RS. Predictors of Poor Outcomes in Critically Ill Adults with Hematologic Malignancy. Can Respir J. 2016;2016:9431385.

88. Freire MP, de Oliveira Garcia D, Garcia CP, Campagnari Bueno MF, Camargo CH, Kono Magri ASG, et al. Bloodstream infection caused by extensively drug-resistant Acinetobacter baumannii in cancer patients: high mortality associated with delayed treatment rather than with the degree of neutropenia. Clin Microbiol Infect. 2016 Apr;22(4):352–8.

89. Faucher E, Cour M, Jahandiez V, Grateau A, Baudry T, Hernu R, et al. Short- and long-term outcomes in onco-hematological patients admitted to the intensive care unit with classic factors of poor prognosis. Oncotarget. 2016 Apr 19;7(16):22427–38.

90. Cornejo-Juárez P, Vilar-Compte D, García-Horton A, López-Velázquez M, Ñamendys-Silva S, Volkow-Fernández P. Hospital-acquired infections at an oncological intensive care cancer unit: differences between solid and hematological cancer patients. BMC Infect Dis. 2016 10;16:274.

91. Reilly JP, Anderson BJ, Hudock KM, Dunn TG, Kazi A, Tommasini A, et al. Neutropenic sepsis is associated with distinct clinical and biological characteristics: a cohort study of severe sepsis. Crit Care. 2016 Jul 18;20(1):222.

92. Greenberg JA, David MZ, Churpek MM, Pitrak DL, Hall JB, Kress JP. Sequential Organ Failure Assessment Score Modified for Recent Infection in Patients With Hematologic Malignant Tumors and Severe Sepsis. Am J Crit Care. 2016 Sep;25(5):409–17.

93. Halpern AB, Culakova E, Walter RB, Lyman GH. Association of Risk Factors, Mortality, and Care Costs of Adults With Acute Myeloid Leukemia With Admission to the Intensive Care Unit. JAMA Oncol. 2017 Mar 1;3(3):374–81.

94. Irie H, Otake T, Kawai K, Hino M, Namazu A, Shinjo Y, et al. Prognostic factors in critically ill patients with hematological malignancy admitted to the general intensive care unit: a single-center experience in Japan. J Anesth. 2017 Oct;31(5):736–43.

95. Ahmed T, Koch AL, Isom S, Klepin HD, Bishop JM, Ellis LR, et al. Outcomes and changes in code status of patients with acute myeloid leukemia undergoing induction chemotherapy who were transferred to the intensive care unit. Leuk Res. 2017;62:51–5.

96. Al-Dorzi HM, Al Orainni H, Al Eid F, Tlayjeh H, Itani A, Al Hejazi A, et al. Characteristics and predictors of mortality of patients with hematologic malignancies requiring invasive mechanical ventilation. Ann Thorac Med. 2017 Dec;12(4):259–65.

97. Demandt AMP, Geerse DA, Janssen BJP, Winkens B, Schouten HC, van Mook WNKA. The prognostic value of a trend in modified SOFA score for patients with hematological malignancies in the intensive care unit. Eur J Haematol. 2017 Oct;99(4):315–22.

98. Russell L, Madsen MB, Dahl M, Kampmann P, Perner A. Prediction of bleeding and thrombosis by standard biochemical coagulation variables in haematological intensive care patients. Acta Anaesthesiol Scand. 2018 Feb;62(2):196–206.

99. Deotare U, Merman E, Pincus D, Kraguljac AP, Croucher D, Kumar V, et al. The utility and safety of flexible bronchoscopy in critically ill acute leukemia patients: a retrospective cohort study. Can J Anaesth. 2018 Mar;65(3):272–9.

100. Maqsood S, Badar F, Hameed A. Characteristics and Outcomes of Patients with Hematological Malignancies Admitted for Intensive Care - a Single Centre Experience. Asian Pac J Cancer Prev. 2017 27;18(7):1833–7.

101. Van de Louw A, Desai RJ, Zhu J, Claxton DF. Characteristics of early acute respiratory distress syndrome in newly diagnosed acute myeloid leukemia. Leuk Lymphoma. 2018;59(10):2369–76.

102. de Vries VA, Müller MCA, Sesmu Arbous M, Biemond BJ, Blijlevens NMA, Kusadasi N, et al. Time trend analysis of long term outcome of patients with haematological malignancies admitted at dutch intensive care units. Br J Haematol. 2018 Apr;181(1):68–76.

103. Tavares M, Lemiale V, Mokart D, Pène F, Lengliné E, Kouatchet A, et al. Determinants of 1-year survival in critically ill acute leukemia patients: a GRRR-OH study. Leuk Lymphoma. 2018;59(6):1323–31.

104. Pastores SM, Goldman DA, Shaz DJ, Kostelecky N, Daley RJ, Peterson TJ, et al. Characteristics and outcomes of patients with hematologic malignancies receiving chemotherapy in the intensive care unit. Cancer. 2018 15;124(14):3025–36.

105. Alp E, Tok T, Kaynar L, Cevahir F, Akbudak İH, Gündoğan K, et al. Outcomes for haematological cancer patients admitted to an intensive care unit in a university hospital. Aust Crit Care. 2018 Nov;31(6):363–8.

106. Al-Zubaidi N, Shehada E, Alshabani K, ZazaDitYafawi J, Kingah P, Soubani AO. Predictors of outcome in patients with hematologic malignancies admitted to the intensive care unit. Hematol Oncol Stem Cell Ther. 2018 Dec;11(4):206–18.

107. Russell L, Haase N, Perner A. Prediction of bleeding by thromboelastography in ICU patients with haematological malignancy and severe sepsis. Blood Coagul Fibrinolysis. 2018 Dec;29(8):683–8.

108. Fassbind P, Jeker B, Mueller BU, Bacher U, Zimmerli S, Endrich O, et al. Improved survival rates of AML patients following admission to the intensive care unit. Leuk Lymphoma. 2019 Oct;60(10):2423–31.

109. Slavin SD, Fenech A, Jankowski AL, Abel GA, Brunner AM, Steensma DP, et al. Outcomes for older adults with acute myeloid leukemia after an intensive care unit admission. Cancer. 2019 01;125(21):3845–52.

110. MacEachern KN, Kraguljac AP, Mehta S. Nutrition Care of Critically Ill Patients with Leukemia: A Retrospective Study. Can J Diet Pract Res. 2019 Mar 1;80(1):34–8.

111. Kondakci M, Reinbach MC, Germing U, Kobbe G, Fenk R, Schroeder T, et al. Interaction of increasing ICU survival and admittance policies in patients with hematologic neoplasms: A single center experience with 304 patients. Eur J Haematol. 2019 Mar;102(3):265–74.

112. Bauer PR, Chevret S, Yadav H, Mehta S, Pickkers P, Bukan RB, et al. Diagnosis and outcome of acute respiratory failure in immunocompromised patients after bronchoscopy. Eur Respir J. 2019 Jul;54(1):1802442.

113. de Oliveira MCF, Ferreira JC, Nassar Junior AP, Dettino ALA, Caruso P. Impact of Urgent Chemotherapy in Critically Ill Patients. J Intensive Care Med. 2020 Apr;35(4):347–53.

114. Camou F, Didier M, Leguay T, Milpied N, Daste A, Ravaud A, et al. Long-term prognosis of septic shock in cancer patients. Support Care Cancer. 2020 Mar;28(3):1325–33.

115. Seong GM, Lee Y, Hong SB, Lim CM, Koh Y, Huh JW. Prognosis of Acute Respiratory Distress Syndrome in Patients With Hematological Malignancies. J Intensive Care Med. 2020 Apr;35(4):364–70.

116. Asdahl PH, Christensen S, Kjærsgaard A, Christiansen CF, Kamper P. One-year mortality among non-surgical patients with hematological malignancies admitted to the intensive care unit: a Danish nationwide population-based cohort study. Intensive Care Med. 2020 Apr;46(4):756–65.

117. Gomez Ravetti C, Ataide TBLS, Barreto LM, Bastos FDL, Gomes AGDR, Detoffol RB, et al. Lung ultrasound is useful in oncohematologic patients with respiratory dysfunction admitted to an Intensive Care Unit (ICU): a pilot study. Med Ultrason. 2020 May 11;22(2):2332.

118. Lemiale V, Pons S, Mirouse A, Tudesq JJ, Hourmant Y, Mokart D, et al. Sepsis and Septic Shock in Patients With Malignancies: A Groupe de Recherche Respiratoire en Réanimation Onco-Hématologique Study. Crit Care Med. 2020 Jun;48(6):822–9.

119. Nassar Junior AP, Trevisani M da S, Bettim BB, Zampieri FG, Carvalho JA, Silva A, et al. Elderly patients with cancer admitted to intensive care unit: A multicenter study in a middle-income country. PLoS One. 2020;15(8):e0238124.

120. Vijenthira A, Chiu N, Jacobson D, Freedman Z, Cheung MC, Goddard S, et al. Predictors of intensive care unit admission in patients with hematologic malignancy. Sci Rep. 2020 Dec 3;10(1):21145.

121. Kalicińska E, Kuszczak B, Dębski J, Szukalski Ł, Wątek M, Strzała J, et al. Hematological malignancies in Polish population: what are the predictors of outcome in patients admitted to Intensive Care Unit? Support Care Cancer. 2021 Jan;29(1):323–30.

122. Ferreyro BL, Scales DC, Wunsch H, Cheung MC, Gupta V, Saskin R, et al. Critical illness in patients with hematologic malignancy: a population-based cohort study. Intensive Care Med. 2021 Oct;47(10):1104–14.

123. McLaughlin K, Stojcevski A, Hussein A, Moudgil D, Woldie I, Hamm C. Patient vital signs in relation to ICU admission in treatment of acute leukemia: a retrospective chart review. Hematology. 2021 Dec;26(1):637–47.

124. Cetintepe T, Cetintepe L, Solmaz S, Calık S, Ugur MC, Gediz F, et al. Determination of the relationship between mortality and SOFA, qSOFA, MASCC scores in febrile neutropenic patients monitored in the intensive care unit. Support Care Cancer. 2021 Jul;29(7):4089–94.

125. Elfassy MD, Ferreyro BL, Rozenberg D, Sklar MC, Mathur S, Detsky ME, et al. Association of Thoracic Computed Tomographic Measurements and Outcomes in Patients with Hematologic Malignancies Requiring Mechanical Ventilation. Ann Am Thorac Soc. 2021 Jul;18(7):1219–26.

126. Park C, Ko UW, Ko RE, Na SJ, Yang JH, Jeon K, et al. Outcomes of extracorporeal membrane oxygenation in adults with active hematologic and nonhematologic malignancy. Artif Organs. 2021 Aug;45(8):E236–46.

127. Aygencel G, Boyacı Dündar N, Türkoğlu M, Yegin ZA, Özkurt ZN, Yağcı AM. Can treating critically-ill haematological malignancy patients in a separate intensive care unit decrease intensive care unit mortality? Turk J Med Sci. 2021 Aug 30;51(4):2095–100.

128. Judickas Š, Stasiūnaitis R, Žučenka A, Žvirblis T, Šerpytis M, Šipylaitė J. Outcomes and Risk Factors of Critically Ill Patients with Hematological Malignancy. Prospective Single-Centre Observational Study. Medicina (Kaunas). 2021 Nov 30;57(12):1317.

129. Lee J, Kim SC, Rhee CK, Lee J, Lee JW, Lee DG. Prevalence and clinical course of upper airway respiratory virus infection in critically ill patients with hematologic malignancies. PLoS One. 2021;16(12):e0260741.

130. Maeng CV, Christiansen CF, Liu KD, Kamper P, Christensen S, Medeiros BC, et al. Factors associated with risk and prognosis of intensive care unit admission in patients with acute leukemia: a Danish nationwide cohort study. Leuk Lymphoma. 2022 Oct;63(10):2290–300.

131. Manjappachar NK, Cuenca JA, Ramírez CM, Hernandez M, Martin P, Reyes MP, et al. Outcomes and Predictors of 28-Day Mortality in Patients With Hematologic Malignancies and Septic Shock Defined by Sepsis-3 Criteria. J Natl Compr Canc Netw. 2022 Jan;20(1):45–53.

132. İleri İ, Özsürekci C, Halil MG, Gündoğan K. NRS-2002 and mNUTRIC score: Could we predict mortality of hematological malignancy patients in the ICU? Nutr Clin Pract. 2022 Oct;37(5):1199–205.

133. Nassar AP, Archanjo LVF, Ranzani OT, Zampieri FG, Salluh JIF, Cavalcanti GFR, et al. Characteristics and outcomes of autologous hematopoietic stem cell transplant recipients admitted to intensive care units: A multicenter study. J Crit Care. 2022 Oct;71:154077.

134. Cantón-Bulnes ML, Jiménez-Sánchez M, Alcántara-Carmona S, Gimeno-Costa R, Berezo-García JÁ, Beato C, et al. Determinants of mortality in cancer patients with unscheduled admission to the Intensive Care Unit: A prospective multicenter study. Med Intensiva (Engl Ed). 2022 Dec;46(12):669–79.

135. Desprez C, Kouatchet A, Marchand T, Mear JB, Tadié JM, Peterlin P, et al. Outcome of patients with newly diagnosed AML admitted to the ICU, including preemptive admission - a multi-center study. Ann Hematol. 2023 Jun;102(6):1383–93.

136. Kundu R, Seeger R, Elfassy MD, Rozenberg D, Ahluwalia N, Detsky ME, et al. The association between nutritional risk index and ICU outcomes across hematologic malignancy patients with acute respiratory failure. Ann Hematol. 2023 Feb;102(2):439–45.
